# Mapping restricted introgression across the genomes of admixed indigenous African cattle breeds

**DOI:** 10.1186/s12711-023-00861-8

**Published:** 2023-12-14

**Authors:** Juliane Friedrich, Richard I. Bailey, Andrea Talenti, Umer Chaudhry, Qasim Ali, Emmanuel F. Obishakin, Chukwunonso Ezeasor, Jessica Powell, Olivier Hanotte, Abdulfatai Tijjani, Karen Marshall, James Prendergast, Pamela Wiener

**Affiliations:** 1 Division of Genetics and Genomics, The Roslin Institute and Royal (Dick), School of Veterinary Studies, University of Edinburgh, Midlothian, UK; 2 Department of Ecology and Vertebrate Zoology, University of Łódź, Łódź, Poland; 3 School of Veterinary Medicine, St. George’s University, St. George’s, Caribbean Grenada; 4 Department of Parasitology, The University of Agriculture Dera Ismail Khan, Khyber Pakhtunkhwa, Pakistan; 5 Biotechnology Division, National Veterinary Research Institute, Vom, Plateau State Nigeria; 6 Department of Veterinary Pathology and Microbiology, University of Nigeria, Nsukka, Enugu State Nigeria; 7 Division of Infection and Immunity, The Roslin Institute and Royal (Dick), School of Veterinary Studies, University of Edinburgh, Midlothian, UK; 8 International Livestock Research Institute (ILRI), Addis Ababa, Ethiopia; 9 School of Life Sciences, University of Nottingham, Nottingham, UK; 10 Centre for Tropical Livestock Genetics and Health (CTLGH), The Roslin Institute, University of Edinburgh, Midlothian, UK; 11 The Jackson Laboratory, Bar Harbor, USA; 12 Centre for Tropical Livestock Genetics and Health (CTLGH), ILRI Kenya, Nairobi, Kenya

## Abstract

**Background:**

The genomes of indigenous African cattle are composed of components with Middle Eastern (taurine) and South Asian (indicine) origins, providing a valuable model to study hybridization and to identify genetic barriers to gene flow. In this study, we analysed indigenous African cattle breeds as models of hybrid zones, considering taurine and indicine samples as ancestors. In a genomic cline analysis of whole-genome sequence data, we considered over 8 million variants from 144 animals, which allows for fine-mapping of potential genomic incompatibilities at high resolution across the genome.

**Results:**

We identified several thousand variants that had significantly steep clines (‘SCV’) across the whole genome, indicating restricted introgression. Some of the SCV were clustered into extended regions, with the longest on chromosome 7, spanning 725 kb and including 27 genes. We found that variants with a high phenotypic impact (e.g. indels, intra-genic and missense variants) likely represent greater genetic barriers to gene flow. Furthermore, our findings provide evidence that a large proportion of breed differentiation in African cattle could be linked to genomic incompatibilities and reproductive isolation. Functional evaluation of genes with SCV suggest that mitonuclear incompatibilities and genes associated with fitness (e.g. resistance to paratuberculosis) could account for restricted gene flow in indigenous African cattle.

**Conclusions:**

To our knowledge, this is the first time genomic cline analysis has been applied to identify restricted introgression in the genomes of indigenous African cattle and the results provide extended insights into mechanisms (e.g. genomic incompatibilities) contributing to hybrid differentiation. These results have important implications for our understanding of genetic incompatibilities and reproductive isolation and provide important insights into the impact of cross-breeding cattle with the aim of producing offspring that are both hardy and productive.

**Supplementary Information:**

The online version contains supplementary material available at 10.1186/s12711-023-00861-8.

## Background

Understanding the genetic basis of population differentiation and identifying genetic barriers to gene flow are important questions in evolutionary biology [1] and have implications for managing breeding of domesticated species. A key issue is what happens to genomic incompatibilities, e.g. allele combinations that result in non-viable or infertile offspring, also referred to as barriers to gene flow, when genetically divergent populations come into contact, and under what circumstances they continue to cause reproductive isolation [2]. Following hybridisation and resulting gene exchange (“introgression”), regions of the genome may differ in terms of both their level of differentiation and their bias in direction of gene flow (“differential introgression”). For example, areas in the hybrid genome may remain differentiated via genomic incompatibilities, maintaining differences between lineages [3]. Differential introgression is a characteristic of hybrid zones: there can be genomic regions with more restricted introgression, such as those containing genetic incompatibilities, and those that are more introgressed in one direction than expected (biased introgression) [3], perhaps representing exchange of adaptations (e.g. [4]). The growing availability of high-density genetic information for hybrid populations brings new opportunities to study the molecular mechanisms of genetic differentiation and genomic incompatibilities at high resolution across the genome, even in non-model organisms. Many domestic livestock species like sheep, cattle or pigs have hybrids or introgressed populations [5] and identifying genomic barriers to gene flow within or between breeds can help to understand and manage breed productivity and diversification in these species. For example, interspecies introgression from mouflon and other wild relatives has been shown to be an important factor for climatic adaptation and pneumonia resistance in sheep [6].

Geographic and genomic cline analyses have been successfully applied in various species to exploit the hybridisation of divergent populations in order to identify restricted introgression [7–12]. The principle of genomic cline analyses is to compare locus-specific admixture in hybrids (the gradient of marker-specific allele frequency changes, i.e. the cline) against the average levels of genome-wide admixture [13–15]. If gene flow is not restricted in either direction, the allele frequency change at that locus will be a linear function of the genome-wide gradient of admixture (the ‘hybrid-index’). A rapid change in allele frequencies relative to the change in hybrid-index can be identified through the cline “steepness” parameter and produces an S-shaped curve, suggesting a “barrier” locus under restricted gene flow (selection against introgression). In addition, the cline “centre” parameter indicates the magnitude of biased introgression in favour of one or the other ancestry [16].

Indigenous African cattle populations, with their complex history [17–20], provide a valuable model to study hybridization and introgression and to identify genetic barriers to gene flow. Cattle (*Bos taurus*) derive from the wild, now-extinct, aurochs (*Bos primigenius*), which had a wide distribution across Europe, Asia, North Africa and the Middle East, and were domesticated in at least two geographic areas: *Bos taurus taurus* (Btt, taurine cattle) in the Middle East (Fertile Crescent) and *Bos taurus indicus* (Bti, indicine cattle) in the Indus Valley (South Asia) [21, 22]. Successive waves of cattle dispersal across the world, including to Africa, occurred via human migration and trading over many centuries. Exact dates vary depending on the region, but it is known that taurine cattle were the first to enter Africa (around 7000–4550 BC [23, 24]), from the Middle East. Indicine cattle have been present in Africa from as early as 2000 BC [25], however, the main migrations from Asia began ~ 700 AD via the Horn of Africa [26]. The most recent waves of cattle migration into Africa started in the twentieth century, with European Btt breeds introduced to improve commercial traits, e.g. milk production [27, 28] and in some cases, Bti. cattle introduced from South America, e.g. [29]. Modern-day African cattle are composed of primarily taurine populations and populations of taurine-indicine hybrids thus making the continent a secondary contact zone for Btt and Bti. Bovine admixture on the African continent is both ancient and recent. For example, major Btt x Bti admixture events in East African cattle can be dated back to ~ 750–1050 years ago [20], while the Rinderpest epidemic in the nineteenth century, which wiped out much of the African cattle population [18], is thought to have been a key factor in the origin of the West African taurine-indicine hybrid zone [30].

Recent studies have characterised signatures of selection in native African cattle [31–36]. In addition, in a comprehensive study on adaptive introgression, Kim et al. [20] have identified genomic regions showing evidence of enhanced taurine or indicine ancestry in these populations, with a number of these regions carrying genes associated with environmental adaptation (e.g. heat tolerance, immune function). However, there has been limited focus on examining the role of restricted introgression in the autosomal genome. Multiple lines of evidence suggest that there may be genomic incompatibilities and reproductive isolation within African cattle genomes, which may lead to restricted introgression. One indication is the strong evidence for reduced fertility in early generation Btt x Bti crossbred cattle (reviewed in [37]). Another factor is that there are no African cattle with very high Bti ancestry; the maximum levels appear to be less than 80% [20], suggesting that there may be limits to introgression into the Btt genomic background. This may partially be due to African cattle being fixed for Btt mitochondria, and recent papers provided evidence of mitochondrial-nuclear incompatibility in African cattle [38, 39].

In this study, we focus on fine-mapping potential genomic incompatibilities and reproductive isolation at high resolution across the genome of indigenous African cattle. To achieve this, we treat indigenous African cattle populations that were sampled from a vast geographic area across the northern half of sub-Saharan Africa as a model of hybrid zones (secondary contact zones), considering taurine and indicine as the ancestral lineages, in order to dissect the outcomes of genetic divergence and subsequent hybridization across the autosomal genome. The advantages of using this unique study system are that much is known about the migration and admixture history of African cattle and furthermore, the African continent encompasses a wide range of environmental conditions, facilitating local adaptation of individual populations. Thus, we expect to capture candidates for restricted gene flow due to either genomic incompatibilities or environmental adaptation. To identify genomic regions of restricted introgression, we used whole-genome sequence data incorporating over 8 million variants and conducted a genomic cline analysis. In this paper, we address the following questions: (1) is the selection of different taurine ancestral populations reflected in signatures of restricted introgression?, (2) are there specific genomic characteristics (e.g. recombination rate, genic variants) that promote restricted introgression?, (3) which regions of the genome show patterns of restricted introgression? and (4) what are the molecular mechanisms contributing to hybrid differentiation? Results from this study will provide general insights into mechanisms of genomic incompatibilities, inform the understanding of population differentiation and may more specifically contribute to conservation and breeding strategies for indigenous livestock in Africa and elsewhere.

## Methods

### Variant calling

Publicly available Illumina sequencing data for 482 cattle genomes representing a wide diversity of global cattle breeds were aligned to the ARS-UCD1.2 cattle reference genome extended with the Y chromosome from the Btau_5.0.1 assembly and processed as described in Dutta et al. [40] and Zhao et al. [41]. Briefly, reads that were aligned to the reference with the BWA-MEM algorithm (v0.7.17) were labelled with GATK [42] PrintReads v4.0.11.0, combined using the BamTools (v2.4.2) software [43] when multiple libraries for a sample were present, and sorted with SAMtools51 ‘sort’ (v1.9) command. Duplicates were marked with the GATK MarkDuplicates (v4.0.11.0) software and base quality score recalibration (BQSR) was performed through BaseRecalibrator and ApplyBQSR by providing the 1000 Bulls genome consortium variants and the variants on the Illumina BovineHD BeadChip. Autosomal variants (single nucleotide polymorphisms (SNPs) and insertions-deletions (indels)) for each sample were called using the standard GATK workflow, calling single sample gVCFs using the HaplotypeCaller software, followed by combining multiple samples using the GenomicDBImport and GenotypeGVCFs tools (v4.0.11.0). The detailed pipeline can be recreated using BAGPIPE (https://bitbucket.org/renzo_tale/bagpipe/).

Variant quality score recalibration (VQSR) from the GATK workflow, a machine learning method that leverages multiple sources of information and parameters to classify the sites into true and false positives (https://gatk.broadinstitute.org/hc/en-us/articles/360035531612-Variant-Quality-Score-Recalibration-VQSR-), was performed using multiple sources (BQSR file from the 1000 Bulls genome project, 24 SNP chip datasets and variants from Ensembl v95 ftp://ftp.ensembl.org/pub/release-95/variation/vcf/bos_taurus/), considering the following parameters at a filtering tranche of 99%: Strand odd ratio (SOR), Fisher strand bias (FS), mapping quality (MQ), quality by depth (QD), mapping quality rank sum test (MQRankSum), the inbreeding coefficient (InbreedingCoeff) and the read position rank sum test (ReadPosRankSum). Indels were filtered using hard filtering recommendations from the GATK developers (https://gatk.broadinstitute.org/hc/en-us/articles/360035531112--How-to-Filter-variants-either-with-VQSR-or-by-hard-filtering) i.e. QD > 2.0, FS < 200.0, ReadPosRankSum > − 20.0, SOR < 10.0.

### Data quality control

From the initial dataset of 482 global cattle genomes, first we removed samples of non-target breeds (e.g. from Australia and America and also non-indicine Asian cattle) and then filtered for at least two samples per breed and a mean sequencing depth of ≥ 8x (n = 302). To further assess sample quality, we filtered for biallelic variants with a call rate ≥ 95% and genotyping quality (QG) > 20. Samples with a call rate < 75% were excluded. The flag “-relatedness2” in vcftools [44] based on the KING method of Manichaikul et al. [45] was used to determine relationship coefficients between pairs of samples. If the relationship coefficient was > 0.177 (1st-degree relationship; as specified in [46]) between a pair of samples of the same breed, one sample was removed. Following this sample-wise quality control, 270 samples comprising 30 breeds remained (see Additional file 1: Table S1), for which the variant-based quality control was repeated, leaving a final dataset of 29,546,954 variants (SNPs and indels). Sample sizes were considerably larger for Holstein Friesian, Boran and N’Dama, representing European taurine, African indicine and African taurine breeds, respectively. Ten genetically representative samples of these populations were selected using the R package ‘corehunter’ [47] using the ‘sampleCore’ function (size = 10, mode = 'fast') to avoid any bias in the subsequent analyses due to this imbalance of sample sizes (final n = 144).

### Determining population structure and admixture

For population structure analyses only, genotype data was pruned using the Plink v1.9 software [48, 49] with the default parameters (‘-indep 50 5 2’) to reduce linkage disequilibrium (LD) between selected variants, resulting in a pruned genotype dataset of 4,878,975 variants. The genomic structure was then analysed using principal component analysis (PCA) in Plink v1.9 [48, 49]. The Admixture software [50] was used for ancestry estimation, where the best number of clusters (*K*) was determined by comparing fivefold cross-validation errors for* K* = 2,…,10.

### Sample grouping for genomic cline analysis

Genomic cline analyses require samples of two divergent ancestral populations (“source” S0 and S1) and their putative hybrids (test samples). For the genomic cline analyses of indigenous African cattle breeds, we pursued two approaches: one approach (“European taurine S1”) with Asian indicine and European taurine samples as ancestral populations S0 and S1, respectively, and all African samples as test samples and a second approach (“African taurine S1”) with Asian indicine and African taurine samples as ancestral populations S0 and S1, respectively, and the remaining African samples as test samples. The aim of this selection of different ancestral populations is to account for the complex history of taurine ancestry in African cattle. Assuming three potential ancestral populations, we selected samples based on admixture proportions from the Admixture ancestry estimations with *K* = 3 (representing Asian indicine, European taurine and African taurine clusters) to group samples based on genomic structure rather than subjective breed labels. Individual Asian indicine samples with a proportion > 0.99 of indicine ancestry were assigned as S0, while individuals with a proportion > 0.99 of European or African taurine ancestry were chosen as S1 for the “European taurine S1” and “African taurine S1” approaches, respectively, with all others classified as test samples (see Additional file 1: Table S2). In total, 141 samples were used in the genomic cline analysis for “European taurine S1” and 87 samples (i.e. excluding European taurine) for “African taurine S1”.

### Genomic cline analysis

Our aim was to identify genomic regions (and associated loci) showing restricted introgression among African cattle. These represent candidate genomic incompatibilities (e.g. [51]) and more generally, genomic regions under some form of selection against foreign ancestry, for example to maintain local adaptation (e.g. to specific environments). A genomic cline describes the gradient of allele frequency changes at a specific locus with respect to the genome-wide admixture proportion or hybrid index (with two ancestral populations, both parameters indicate the proportion of the genome inherited from one ancestral population—‘S1’ in this case). Two locus-specific parameters are estimated, one that indicates the gradient of change in allele frequency and can be used to determine regions with restricted gene flow (selection against introgression), and a second indicating the extent of bias in the direction of introgression favouring one or the other ancestor.

The genomic cline analysis was performed using the R package ‘gghybrid’ [16, 52], which has been applied in a number of other studies [53–55]. This package uses Fitzpatrick’s logit-logistic genomic cline function [13] and estimates parameters *v* (locus-specific cline steepness relative to genome-wide hybrid index) and *centre* (the genome-wide hybrid index at which locus-specific allele frequency is halfway between those of the ancestral populations S0 and S1). The parameter *v* is always positive and *v* > 1 indicates a steep cline and hence restricted gene flow, (the higher *v,* the more restricted the gene flow in both directions across the cline *centre*), while *v* < 1 indicates a shallow cline (allele frequency changes at a slower rate than the change in genome-wide hybrid index). The second parameter, cline centre (*c*), indicates a bias of gene flow in favour of one or the other ancestral allele (biased introgression) and ranges from 0 to 1 depending on which ancestry is favoured (e.g. cline centres closer to 0 indicate a stronger bias in favour of introgression of the S1 allele into the S0 genomic background), with a value of 0.5 indicating no bias.

For hybrid index and genomic cline estimation, gghybrid applies a Bayesian Markov chain Monte Carlo (MCMC) method. Hybrid index (h-index, ranging from 0 for pure S0 to 1 for pure S1) was estimated using the gghybrid function ‘esth’ on the pruned dataset (4,878,975 variants) rather than the full set of variants because it provides sufficient genome-wide variant coverage while reducing computational time and avoiding statistical correlations among loci (patterns of introgression could be biased by variants in linkage disequilibrium). Variants were further filtered using the “AF.CIoverlap = FALSE” option in the ‘data.prep’ function of ‘gghybrid’, to include only those with high-confidence allele frequency differences between ancestries. We used a burn-in of 1000 iterations (“burnin = 1000”) and 1000 subsequent iterations (“nitt = 2000”), following a comparison for *Bos taurus* autosome (BTA) 1 with h-index estimates based on larger numbers of iterations (nitt = 5000, burnin = 2000), which indicated that the smaller number of iterations was sufficient to effectively sample the posterior distribution.

Genomic cline analysis was performed using the function ‘ggcline’ on the unpruned genotype dataset, again filtered using “AF.CIoverlap = FALSE” within ‘data.prep’, to maximize resolution across the genome. Calculating variant-wise rather than window-wise parameters of restricted introgression is consistent with other studies on genomic cline analysis and enables us to test different variant-based hypotheses (e.g. effect of variant properties on restricted introgression). S0 and S1 individuals were included in the cline analysis (by setting “include.Source = TRUE”). In total, 8,245,162 variants remained after filtering for the European taurine S1 and 5,899,460 for the African taurine S1 cline analyses. After testing different numbers of iterations, we ran ‘ggcline’ with burnin = 2000 and nitt = 5000, as this was sufficient for effective posterior sampling and convergence. The R package gghybrid jointly estimates cline steepness (*v*) and centre (*c*) on a latent scale as ln(*v*) and logit(*c*), to ensure a posterior bivariate normal distribution per locus, and provides locus-wise posterior means on the latent and original scale, latent scale variances (and covariance), Bayesian p-values, and original-scale 95% credible intervals for both statistics. Simulations have shown that the change in p-values is not fully consistent with the magnitude of parameter deviation from the null value, particularly for parameter *v* [16], and the “widely applicable information criterion” (waic) statistic was recommended instead to identify variants with significant deviations from the null hypothesis [16]. Therefore, to identify variants with significantly steep clines, we used the preferred waic method. We repeated cline estimation with ‘ggcline’, fixing cline steepness to *v* = 1 (reduced model) for all loci. Then, the ‘compare.models’ function was applied to calculate “Δwaic” (waic difference between full and reduced models) for each variant. Negative “Δwaic” indicates stronger support for the full model and hence a statistical deviation in *v* from the genome-wide hybrid index.

### Identifying variants and candidate regions showing restricted introgression

The main focus of this study was the dissection of restricted introgression in the African cattle genome by identifying variants with restricted introgression (steep clines) with ln(*v*) > 2.3, which corresponds to very steep clines of *v* > 10. Following this filtering step, the parameter Δwaic (described above) was used to determine significantly steep clines; the threshold for significantly steep clines was Δwaic < −10 (for comparison, a threshold of Δwaic < −2 used in simulation studies resulted in about 5% false positives out of thousands of tested loci) [16]. We chose this very conservative threshold to decrease the chances of false positive variants: real data may not fit the cline model as well as simulated data and some variants evolving by drift rather than selection might by chance pass the lower statistical threshold. Variants passing these two filter criteria are referred to as “variants with significantly steep clines” (steep cline variants; SCV) in the following. To analyse variants with significantly steep clines and biased introgression, we grouped SCV based on their cline centre (*c*) to indicate indicine biased (*c* ≥ 0.55), taurine biased (*c* ≤ 0.45) and unbiased introgression (*c* > 0.45 and < 0.55). We refer to variants with both steep clines and biased introgression (i.e. with *c* ≤ 0.45 or ≥ 0.55) as “variants with differentiated introgression”.

Regions of restricted introgression may be clustered within the genome, for example due to limited recombination or clustering of genes under selection. To examine this, in addition to determining individual SCV, we identified candidate restricted introgression regions by pooling SCV within 50 kb of at least one other SCV. More specifically, a region was identified if the distance between consecutive SCV was less than 50 kb: the start point was the position of the first SCV, and the region continued as long as consecutive SCV were within 50 kb of each other, ending when the distance between consecutive SCV was larger than 50 kb (the end point was the position of the last SCV). The same method was applied to SCV that were in the same cline centre group (indicine biased, taurine biased or unbiased) to determine candidate genomic regions of differentiated introgression. In both cases, to minimise the chance of false positives, we eliminated candidate regions with fewer than ten variants.

Positions of SCV and regions of restricted introgression were mapped against the ARS-UCD1.2 *Bos taurus* genome assembly [56]. Enrichment analyses for the respective gene sets (i.e. genes harbouring SCV and genes within regions of restricted introgression) were conducted in Enrichr [57, 58]. To test for the association between restricted introgression and reproduction, we used Cattle GTEx data [59]) and limma, an R package that analyses gene expression microarray data [60], to identify genes that are overexpressed in reproductive tissues (ovary and testis) compared to other tissues.

### Effect of variant attributes on cline steepness

We used the Ensembl Variant Effect Predictor (VEP) tool [61] to assign effects to variants (SNPs and indels). Variants in coding and non-coding regions of the genome were annotated, based on the ARS-UCD1.2 reference genome (VEP (v102)), and only the most severe consequence per variant was extracted (‘-most_severe’ flag). A linear regression model (y ~ x) was fitted in R (‘lm(y ~ x)’) with y = ln(*v*) and x = effect category (described here http://www.ensembl.org/info/genome/variation/prediction/predicted_data.html) to test for relationships between variant effect and cline steepness. Underrepresented categories (less than 1000 variants) were removed from the comparison. The tool SnpSift [62] was used to assign types (SNP or indel) to variants. The significance of differences (p) between the average cline steepness ln(*v*) for variant types was tested by a Mann–Whitney–Wilcoxon test.

### Differentiation of variants among African cattle breeds

Genetic differentiation among cattle breeds may only be partially related to hybridisation. For all African cattle breeds with at least seven samples (see Additional file 1: Table S1), we determined the genetic differentiation of variants among breeds without reference to the h-index using an across-population fixation index (F_ST_). In Plink v1.9 [48, 49], Weir’s F_ST_ was computed for multiple subpopulations (specified by ‘-within') using the flag ‘-fst'. Negative F_ST_ values were set to zero. The association between variants grouped according to their F_ST_ and cline steepness was tested in an analysis of variance (ANOVA) for the European and African taurine S1 approaches, in order to test the extent to which breed differentiation was linked to restricted introgression.

### Relationships between recombination rate and allele frequency patterns

Regions of low intra-specific recombination may be highly differentiated among populations and harbour an excess of genomic incompatibilities. In order to relate the results for cline steepness and within-Africa genetic differentiation (F_ST_) to patterns of taurine recombination, we used estimates of recombination rate from single sperm sequencing of two Holstein bulls [63], which were presented as the number of crossover events for each 1-Mb region across the genome. We divided the genome into three region categories: high recombination, low recombination and all other regions. We defined regions of high recombination as the hotspots, defined by Yang et al. [63] (segments with a recombination rate of 2.5 × standard deviation greater than the mean), that were shared by the two sires (Table S10 in Yang et al. [63]). We defined regions of low recombination as those that showed no recombination events in blocks of three or more 1-Mb segments in either sire (“coldspots”) (results shown in Fig. 2C in Yang et al. [63] and details provided by the authors). Linear regression with ln(*v*) and F_ST_ as response variables were performed to test for differences between the three categories of genomic regions.

## Results

### Genetic structure

**Fig. 1:**
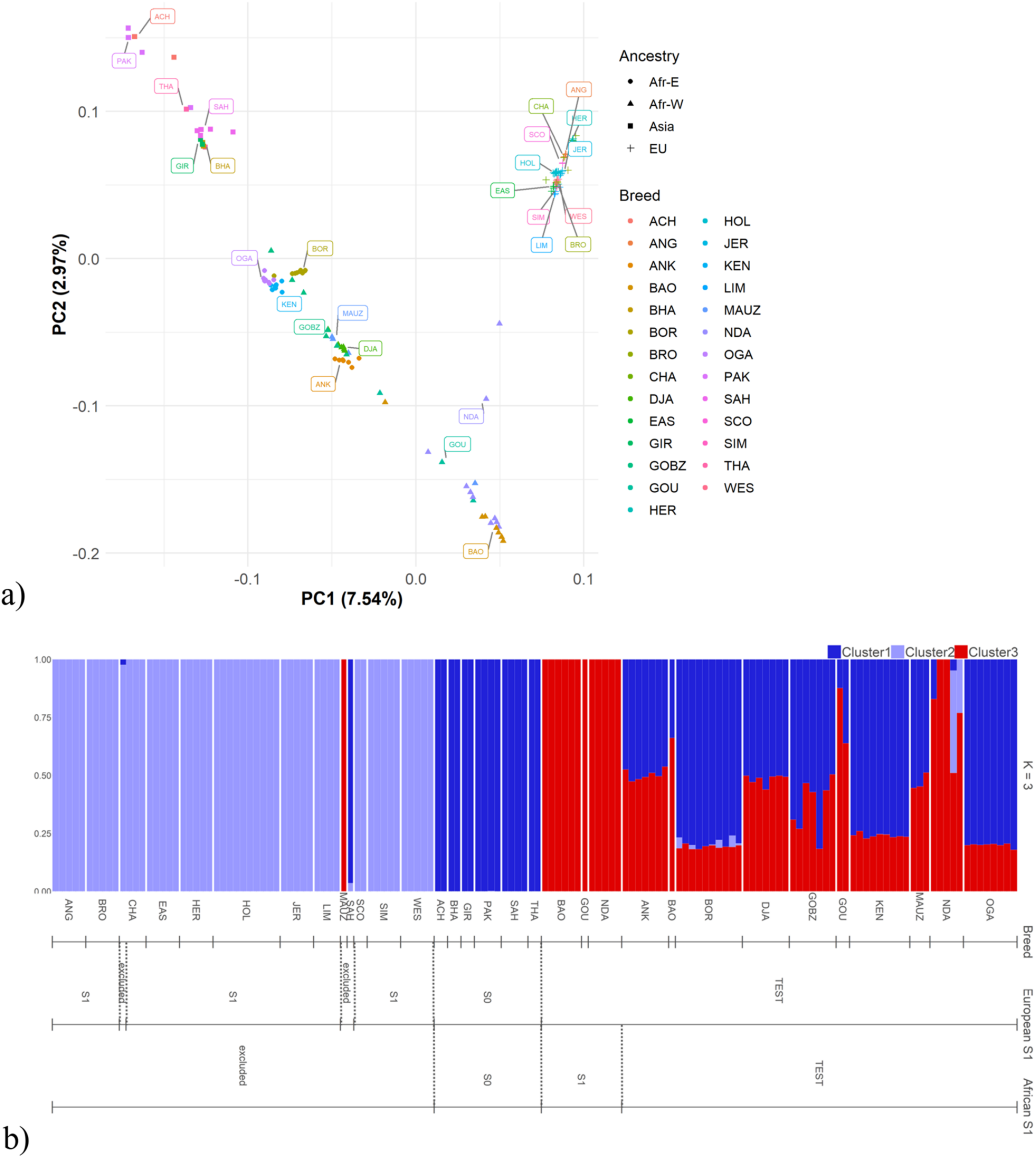
Principal component analysis (PCA) and admixture analysis of African, European and Asian cattle. Eigenvectors for the first two principal components are plotted and the variances explained by the principal components are given in parentheses (**a**). Proportion of genetic admixture for K = 3 of 144 African, European and Asian cattle samples (**b**). Breed abbreviations: ACH, Achai; ANG, Angus; ANK, Ankole; BAO, Baoule; BHA, Bhaghnari; BOR, Boran; BRO, Brown Swiss; CHA, Charolais; DJA, Djakkore; EAS, Eastern Finncattle; GIR, Gir; GOBZ, Zebu Gobra; GOU, Gourounsi; HER, Hereford; HOL, Holstein; JER, Jersey; KEN, Kenana; LIM, Limousine; MAUZ, Zebu Maure; NDA, N’dama; OGA, Ogaden; PAK, other Pakistani breeds (Cholistani, Dhanni, Gabraali, HisarHyana); SAH, Sahiwal; SCO, Scottish Highland; SIM, Simmental; THA, Tharparkar; WES, Western Finncattle

To determine the genetic structure of the dataset, principal component and Admixture analyses were performed on the pruned genomic dataset of the 144 samples that passed the initial sample-wise quality control. The PCA revealed that 7.5% of the genetic variation observed in the data can be attributed to *indicine-taurine* differentiation (PC1) (Fig. 1a), where Asian indicine samples and European taurine samples are located at opposite ends of PC1. Furthermore, PC2 (accounting for 3.0% of the genetic variance) discriminates between European taurine and African taurine samples and also between Asian indicine and African samples, with European and Asian samples having positive scores for PC2 and African samples having negative scores. The Admixture analysis of 270 samples was performed for *K* = 2,…,10 clusters. Based on the lowest cross-validation error and number of iterations, *K* = 3 clusters (ancestries) best explained the observed variance in the genomic data. For *K* = 3, a priori information of breeds with the highest membership coefficients for the respective ancestries indicate that the clusters can be labelled as “Asian indicine” (cluster 1, dark blue), “European taurine” (cluster 2, light blue) and “African taurine” (cluster 3, red) **(**Fig. 1b). Samples with high proportions of the “African taurine” cluster were predominantly found in West African cattle populations. Samples for subsequent cline analyses were selected based on K = 3. Samples with membership coefficients > 0.99 for the respective clusters were grouped into the S0 (indicine) and two different S1 (taurine) ancestries (see “Methods” for details).

### Hybrid index

**Fig. 2:**
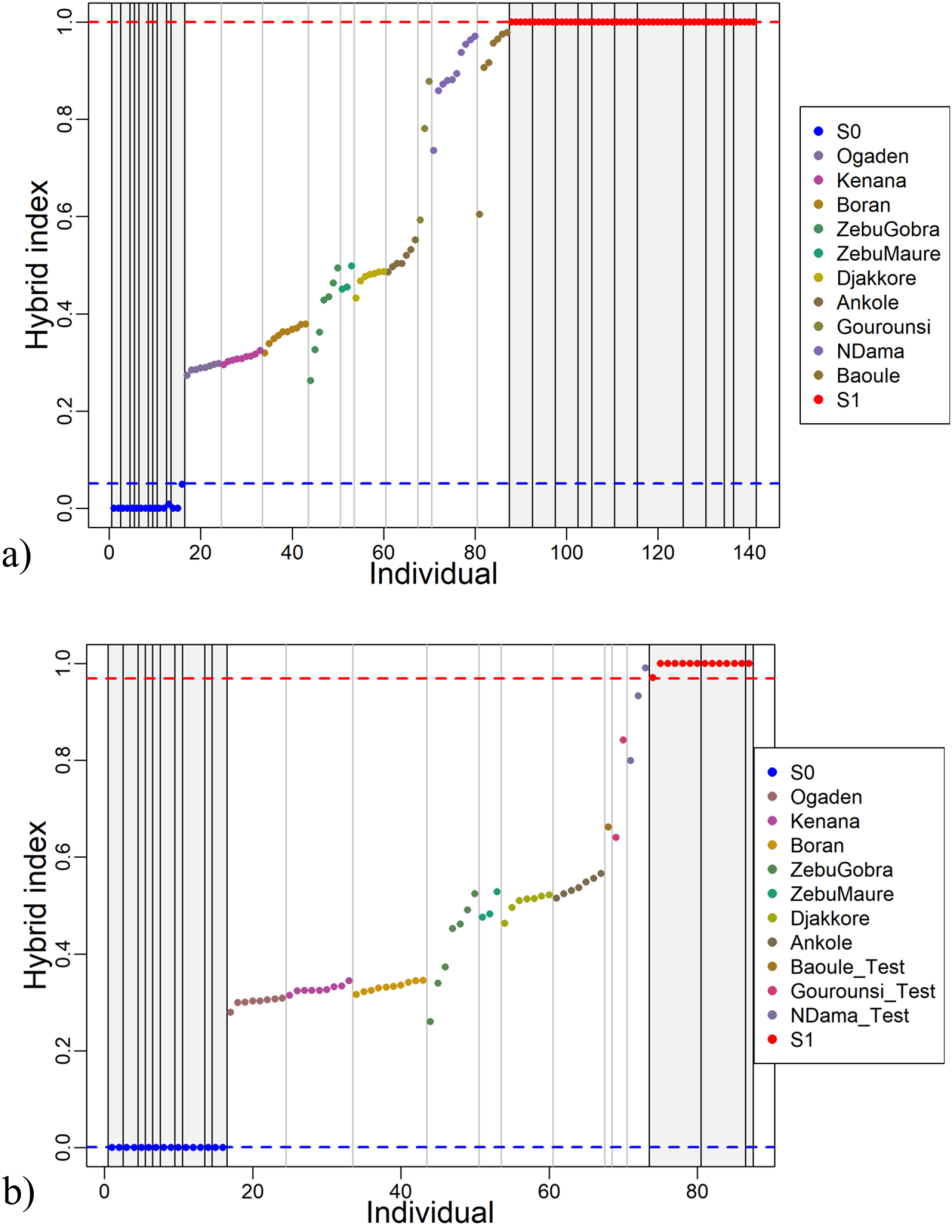
Distribution of hybrid-indices of admixed African cattle. Hybrid-indices are shown for (**a**) European taurine S1 and (**b**) African taurine S1. Admixed African samples are coloured according to their breed and ordered with increasing h-index, with S0 = Asian indicine ancestral population and S1 = African or European taurine ancestral population. For (**b**) S1 consisted of a subset of samples from the African Baoule, Ndama and Gourounsi breeds. These breeds also had samples in the test set (labelled by “_Test”)

We applied two approaches for the genomic cline analysis to dissect the taurine influence on hybrid populations: one where European *Bos taurus* was considered as the taurine ancestry (“European taurine S1” approach) and one where African *Bos taurus* was considered as this ancestry (“African taurine S1” approach). Asian indicine represents the S0 ancestry in both approaches. Using pruned genotype data, the proportion of the genome originating from S1 (h-index) was calculated for each individual, including S0 and S1 samples (Fig. 2). For both approaches, most test samples had greater estimated Asian indicine than taurine ancestry (h-index < 0.5), but no samples had very high proportions of indicine ancestry (all h-indices were higher than 0.2), indicating a lack of samples of relatively pure indicine background. Breeds with the lowest h-indices (greatest indicine ancestry) were the East African Ogaden, Kenana and Boran breeds, while the West African N’Dama, Baoule and Gorounsi test samples had the highest h-indices (greatest taurine ancestry). Zebu Gobra samples showed the largest variation in h-indices for both approaches. Test samples with approximately equal proportions of indicine and taurine ancestry (h-index ~ 0.5) comprised Ankole for the European taurine S1 approach and Djakkore for the African taurine S1 approach.

### Genomic cline analysis for identification of restricted introgression

After excluding variants with an overlap in 95% credible intervals of ancestral allele frequency differences, we retained 8,245,114 variants for the European taurine S1 and 5,899,460 for the African taurine S1 from the unpruned genotype dataset (5,198,538 variants overlapped). In total, 15,029 and 9682 variants, in the European taurine S1 and African taurine S1, respectively, had significantly steep clines based on stringent and conservative filtering (ln(*v*) > 2.3 and Δwaic < -10), indicating restricted introgression (see Additional file 2: Fig. S1). In total, 2483 variants with significantly steep clines (SCV) overlapped between the two approaches, which corresponds to 16.5% and 25.6% of SCV in the European taurine S1 and African taurine S1 approaches, respectively. The correlation between ln(*v*) for the two approaches across the 2483 variants was 0.79. The largest numbers of overlapping SCV were found on three chromosomes: BTA21 (n = 339), BTA5 (n = 281) and BTA14 (n = 239). The European taurine S1 analyses resulted in significantly steeper clines (mean ln(*v)* = 0.20) than the African taurine S1 approach (mean ln(*v)* = -0.62) (see Additional file 2: Fig. S2). The correlation between ln(*v)* was 0.65 for the cline steepness of all overlapping variants between European taurine S1 and African taurine S1 approaches.

**Fig. 3:**
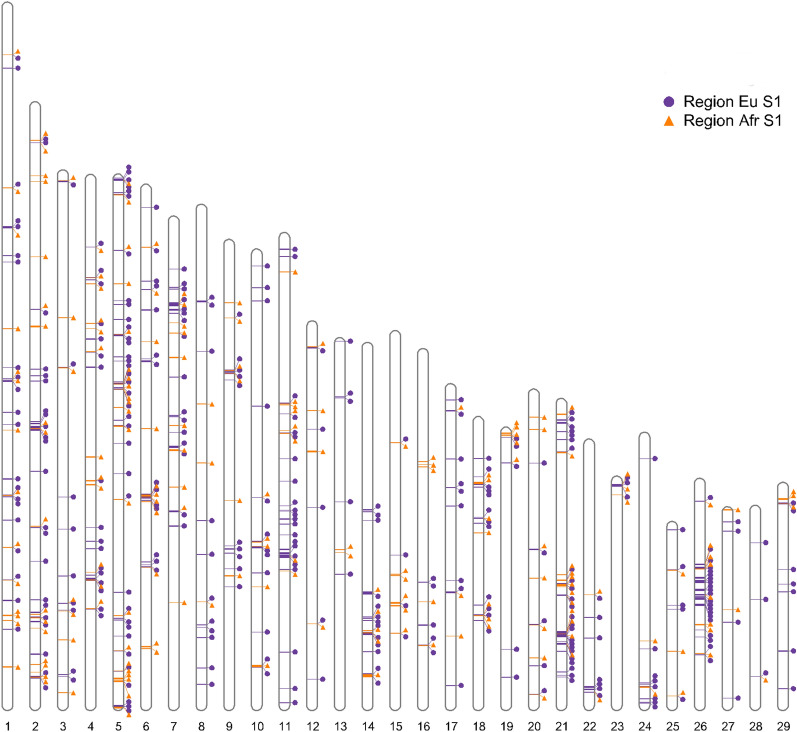
Candidate regions for restricted introgression. Variants with significantly steep clines (SCV) were assigned to candidate regions when these variants were within 50 kb of each other, and the candidate region included at least ten variants. Genomic locations of candidate regions for restricted introgression are shown for European taurine S1 (blue circles) and African taurine S1 (orange triangles)

To determine genomic regions with strong signals of restricted introgression, we grouped SCV into candidate restricted introgression regions where variants were within 50 kb of each other. We identified 335 candidate regions for restricted introgression, each containing at least 10 SCV in close proximity, for European taurine S1 and 194 candidate regions for African taurine S1 (see Additional file 3: Table S3 and Fig. 3). These restricted introgression regions ranged in length from 0.9 to 725 kb (European S1) and from 2.8 to 566 kb (African S1). Sixty-six candidate regions for restricted introgression overlapped between the approaches, which corresponds to 20% of the European taurine S1 and 34% of the African taurine S1 candidate regions.

### Association between variant attributes and restricted introgression

**Fig. 4:**
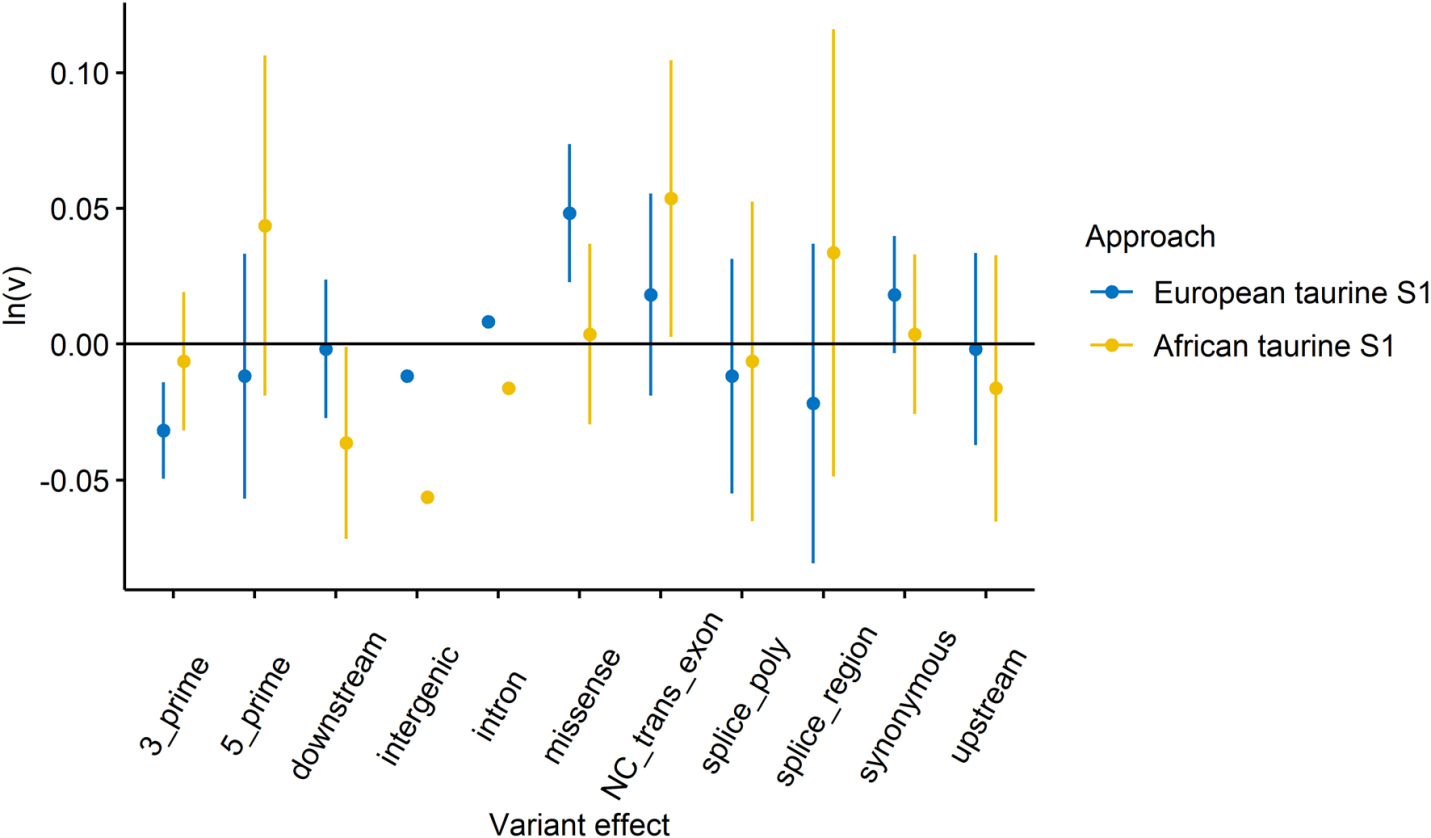
Association between variant effect and cline steepness (v). Variant effects predicted by the VEP tool [61] were tested for their effect on *v* using a linear regression (y ~ x) where y = ln(*v*) and x = effect category. The plot shows the estimated mean effect size and 95% confidence intervals for each effect category. Higher estimated ln(*v*) above zero indicates stronger evidence for restricted introgression

We fitted a linear model to test for associations between variant effects and cline steepness, *v*. For both approaches, there was a significant association between variant effect and ln(*v)* (European taurine S1: F-statistic = 53.0, p-value < 2.2e−16; African taurine S1: F-statistic = 129.9, p-value < 2.2e−16). For the European taurine S1, “missense variants” (which cause changes in amino acid composition) had the steepest clines and for the African taurine S1, “non-coding transcript exon variants” had the steepest clines, and for every effect, the standard errors were lower for the European taurine S1 (Fig. 4 and see Additional file 4: Table S4).

The tool SnpSift [60] was used to assign types (SNP or indel) to variants. For both taurine S1 approaches, 92% of loci were SNPs and 8% were indels. Indels had significantly steeper clines compared to SNPs for both approaches: for the European taurine S1 approach, mean ln(*v*) was 0.24 ± 1.35 for indels and 0.19 ± 1.32 for SNPs (p-value < 2e−16) and for the African taurine S1 approach, mean ln(*v*) was −0.60 ± 1.57 for indels and −0.63 ± 1.55 for SNPs (p-value < 2.9e−15).

### Genes associated with restricted introgression

SCV were mapped against the ARS-UCD1.2 assembly. For the European taurine S1, 5599 SCV (37.3% of all SCV) were located within genes (1077 total genes) and for the African taurine S1, 2161 SCV (22.3% of all SCV) were within genes (868 total genes). In contrast, for the unfiltered dataset, only 12.0% and 12.5% of all variants were located within genes for the European taurine S1 and African taurine S1, respectively. In total, 245 of these genes overlapped between the two approaches. For the European taurine S1, the five genes with the largest number of SCV were *BTRC* (BTA26; 121 SCV), *TMEM117* (BTA5; 115 SCV), *BABAM2* (BTA11; 110 SCV), *SNX29* (BTA25; 107 SCV) and *GEMIN5* (BTA7; 88 SCV). For the African taurine S1, the five genes with the largest number of SCV were *MMP16* (BTA14; 232 SCV), *EIF3E* (BTA14; 68 SCV), *TOGARAM1* (BTA21; 35 SCV), *PCDHCGC3* (BTA7; 35 SCV) and *ATP8B1* (BTA24; 34 SCV). The genes with the largest number of SCV overlapping between the approaches were *EIF3E* (67 SCV), *TOGARAM1* (23 SCV) and *GPC6* (22 SCV). Enrichment analyses for the respective gene sets were conducted in Enrichr [57, 58]. No individual gene ontology (GO) biological processes were significantly enriched after correction for multiple testing for European taurine (see Additional file 2: Fig. S3), while “cell–cell adhesion via plasma-membrane adhesion molecules” and “regulation of synapse assembly” were significantly enriched for the African taurine S1 (see Additional file 2: Fig. S4). Significantly steep clines located within genes that overlapped between the approaches encompassed a gene set of 245 genes, which were significantly enriched for the GO biological process “cellular response to low-density lipoprotein particle stimulus” (see Additional file 2: Fig. S5).

We also extracted genes located within the candidate genomic regions of restricted gene flow to capture potential candidate genes near (but not necessarily directly overlapping) SCV (see Additional file 3: Table S3). In total, 406 genes were located within the 335 candidate regions of restricted gene flow identified in the European taurine S1. The steepest clines were found for variants located within the region REG_eu917 on BTA6 (average ln(*v)* = 4.3), which contained only the *ODAM* gene. The longest candidate regions for restricted introgression were REG_eu960 on BTA7 (including 27 genes) and REG_eu1673 on BTA14 (including the *EMC2* and *EIF3E* genes). REG_eu1673 also had the largest number of SCV. The candidate region with the second largest number of SCV was REG_eu1749 (BTA15), which contained only the *FSHB* gene.

In total, 248 genes were located in the 194 candidate regions of restricted gene flow identified by the African taurine S1 approach. Of these, 126 overlapped with genes in candidate regions from the European taurine S1. The region with the steepest clines (average ln(*v)* = 3.9) was detected on BTA6 (REG_afr742, including the *CRMP1, EVC* and *EVC2* genes, and 2 Mb upstream of the REG_eu917 region, mentioned above). The longest candidate region was on BTA14 (REG_afr1419), which overlapped with a region detected by the European taurine S1 approach (REG_eu1705) (including the *MMP16* gene).

For the African taurine S1 approach, we found significant enrichment (p-value = 0.001) for genes located in genomic regions with restricted introgression (Additional file 3: Table S3a, b) in the set of genes that are over-expressed in ovary tissue, but there were no significant enrichments for genes over-expressed in testis tissue or for either ovary or testis tissue for the European taurine S1 approach.

### Association between cline steepness and biased introgression

We considered cline steepness and centre together, using deviations from the neutral cline centre (0.5) as an indicator for biased introgression. For both the European and African taurine S1 approaches, the largest number of SCV (41% and 43%, respectively) showed no bias in cline centre (*c* = 0.45–0.55) (Table 1). However, only 11% and 8% of variants, respectively, showed no bias in introgression for the unfiltered datasets including all variants (i.e. no filtering for significantly steep clines) (Table 1). The difference in frequencies of variants within cline centre groups between the filtered (SCV only) and unfiltered datasets was highly significant (European taurine S1: *Χ*^2^ = 13,151, df = 2; p < 0.0001; African taurine S1: *Χ*^2^ = 17,118, df = 2; p < 0.0001), indicating an enrichment of variants with restricted gene flow and unbiased introgression.

**Table 1:** Direction of restricted gene flow

Cline centre	European taurine S1	African taurine S1
Indicine biased: ≥ 0.55	4869 (3,256,939)	4082 (2,040,189)
Unbiased: > 0.45 and < 0.55	6157 (930,332)	4123 (444,122)
Taurine biased: ≤ 0.45	4003 (4,057,891)	1477 (3,415,149)

The number of variants with significantly steep clines (SCVs) are shown in cline centre groups, indicating trends of ancestries. Variants with cline centre ≤ 0.45 indicate biased gene flow favouring the taurine allele, variants with cline centre ≥ 0.55 indicate biased gene flow favouring the indicine allele and variants with cline centre > 0.45 and < 0.55 indicate unbiased introgression. The number of variants within the groups without filtering for variants with restricted introgression is given in parentheses

We also grouped nearby SCV (within 50 kb) that were in the same cline centre group (indicine biased, taurine biased or unbiased) into candidate regions for restricted introgression (see Additional file 5: Table S5) and assigned genes to these regions. Out of 322 and 170 genomic regions, for the European taurine S1 and African taurine S1 approaches, respectively, the smallest numbers of regions (73 and 21) were identified for variants with taurine bias (*c* < 0.45). More regions (93 and 67, for the European taurine S1 and African taurine S1 approaches, respectively) were detected for variants with indicine bias (*c* > 0.55). However, most regions for both approaches had no shifted centre (156 and 82 regions, respectively) indicating no biased introgression for regions with restricted gene flow.

### Restricted introgression and breed differentiation

**Fig. 5:**
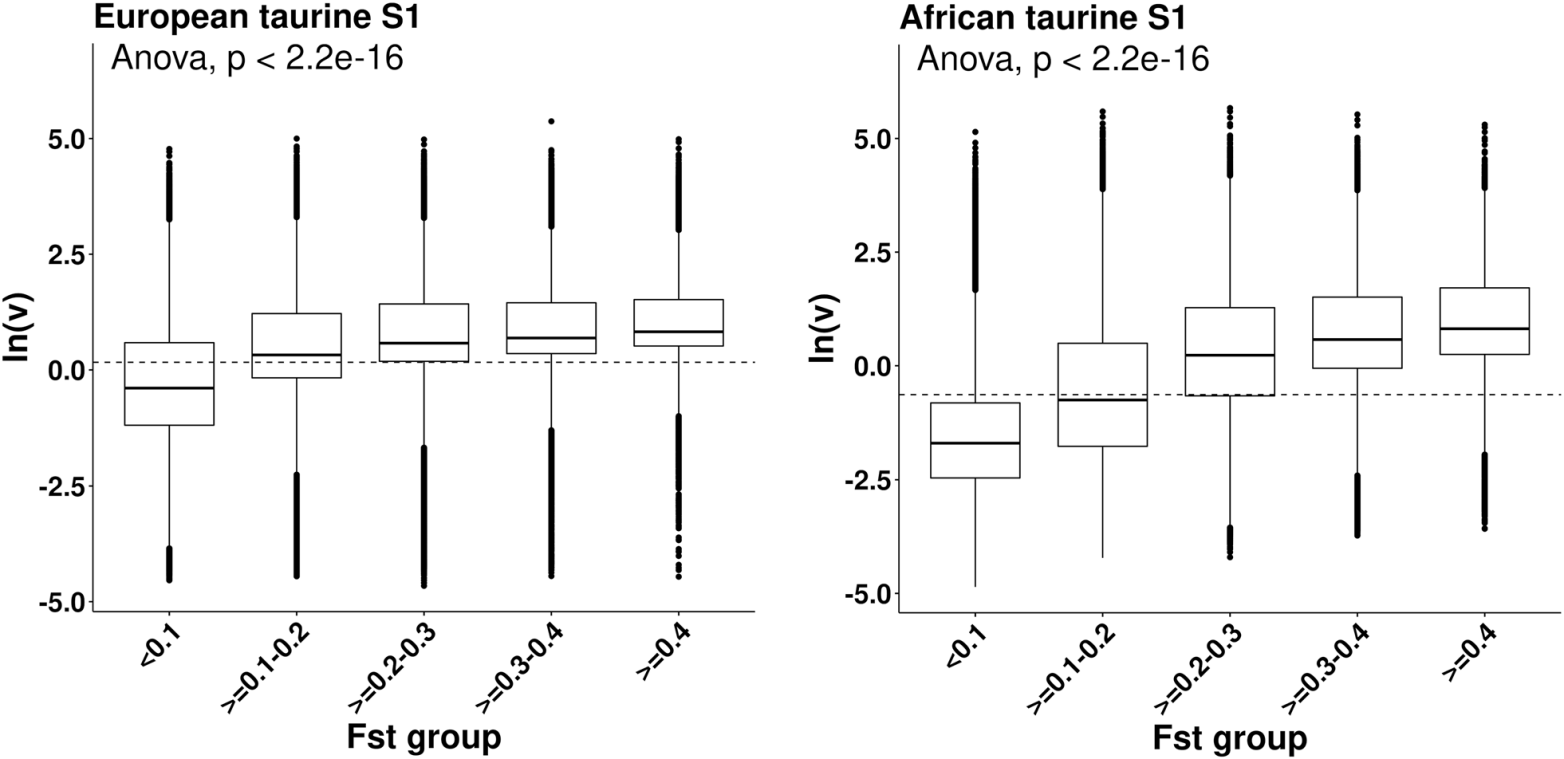
Mean cline steepness (v) grouped by genetic differentiation of variants among African breeds. Mean ln(*v*) is shown in across-population F_ST_ groups computed using all African breeds with at least seven samples. Mean ln(*v*) was compared between F_ST_ groups using ANOVA

An across-population fixation-index (F_ST_) statistic was calculated using all African breeds with more than seven samples to determine between-breed genetic differentiation of variants. Cline steepness ln(*v*) increased significantly with higher values of F_ST_ (Fig. 5) as tested in an ANOVA, and this was especially clear for the African taurine S1. The correlation between F_ST_ and ln(*v*) was moderate to high with 0.39 for the European taurine S1 and 0.51 for the African taurine S1.

### Relationships between recombination rate and allele frequency patterns

Eight regions (encompassing 12 Mb, across seven chromosomes) qualified as high recombination regions (“hotspots”). Twenty-seven low recombination regions (“coldspots”) were found as defined in the Methods (encompassing 94 Mb, across 18 chromosomes). The remainder of the genome was considered “other.”

**Fig. 6:**
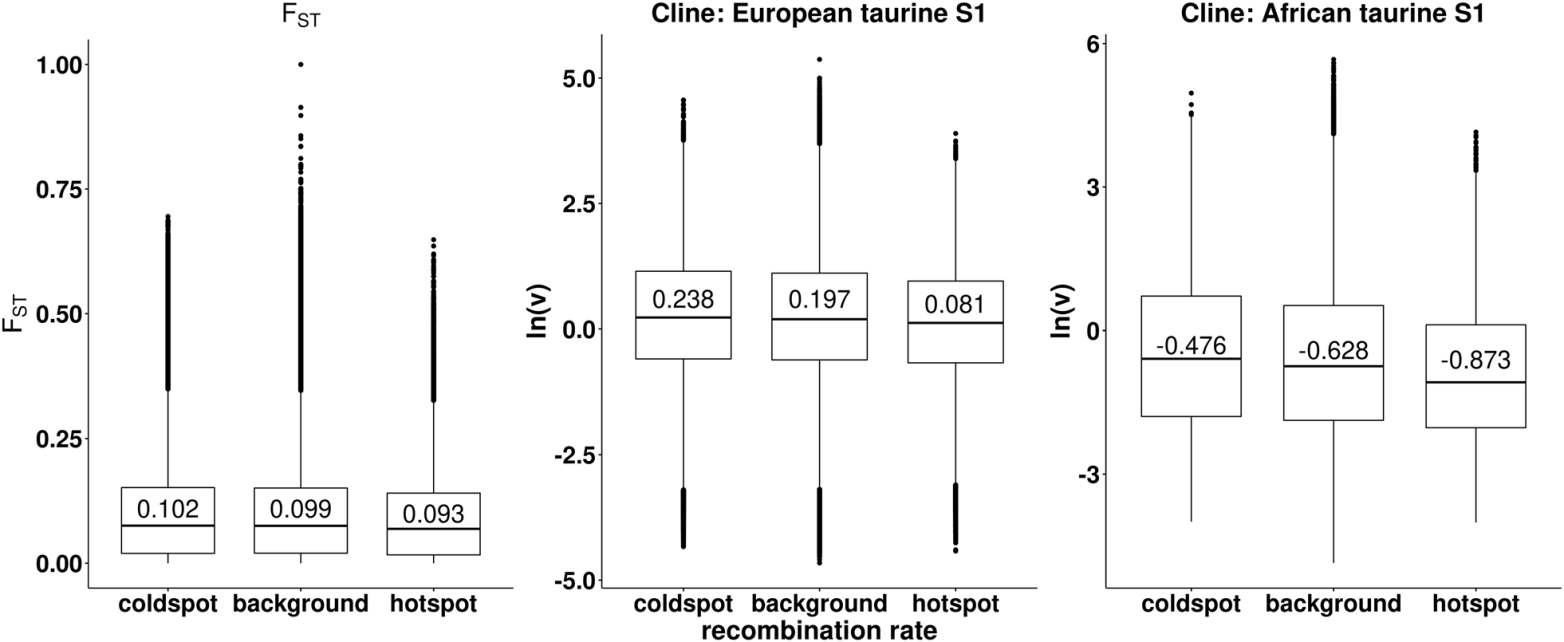
Association between allele frequency patterns and recombination

There were significant differences between recombination categories of the genome in terms of allele frequency patterns for both the European taurine S1 and African taurine S1 approaches (Fig. 6) such that cline steepness estimates and F_ST_ values were greatest in coldspots and lowest in hotspots, with other regions falling in between (see Additional file 6: Table S6). The differences between other and hotspot regions were greater than those between other and coldspots regions.

## Discussion

In this study, we analysed indigenous African cattle breeds as a model of hybrids, considering taurine and Asian indicine samples as the ancestral populations, in order to dissect the forces that affect genetic divergence and hybridization. We used whole-genome sequence data to perform a genomic cline analysis with the aim of identifying regions that showed patterns of restricted introgression.

### Genetic structure and African cattle breed characterisation

Population structure analyses (PCA and Admixture) that were performed to initially characterise the dataset showed a clear separation of the cattle populations. In our analyses, PC1 separated taurine and indicine cattle breeds while PC2 separated European and Asian cattle from African taurine cattle, indicating clusters of European taurine, Asian indicine, admixed African cattle breeds and African taurine breeds, which is consistent with previous studies based on much less dense marker arrays [19, 64, 65]. The cluster number with the lowest cross-validation error in the Admixture analysis (K = 3) revealed genetic heterogeneity in the African breeds, with a wide range of admixture proportions of taurine and indicine backgrounds and dominance of African, rather than European, taurine ancestry. European taurine ancestry was only detectable for a few N’Dama individuals. As a general pattern, we found that the proportions of indicine ancestries decreased from East to West Africa, which is also consistent with previous studies [66, 67].

The hybrid-index (h-index) represents another approach to characterise hybrid genetic structure and thus these results were consistent with those from the Admixture analysis. One key finding in the genetic structure analysis was that the indicine ancestry was dominant in the analysed cattle; most cattle had higher proportions of indicine than taurine ancestry (h-index < 0.5). Yet, we were unable to identify samples with a very high or entirely indicine composition (h-index < 0.2). This lack of breeds with extreme indicine composition may reflect a sampling bias in our study, but it is more likely that no breeds of pure indicine indigenous cattle currently exist in Africa, as previous studies have also failed to identify these [19, 20]. The reason behind this still remains unclear, but one hypothesis is that mitonuclear incompatibilities exist, which restrict indicine gene flow: functional mismatches between the pure taurine mitochondrial genome and indicine genotypes at corresponding genes in the nuclear genome may cause barriers to indicine gene flow into the taurine background. Identifying genomic regions with disproportionally high taurine ancestry across all breeds, which contribute to the observed “minimum taurine ancestry” of at least 20% in all samples, is an important target for further studies.

### Factors influencing restricted introgression

We applied two genomic cline models: one investigating clines between European taurine and Asian indicine ancestries and one between African taurine and Asian indicine ancestries. Previous studies (e.g. [19, 68]) have shown a clear separation between these three clusters (European taurine, Asian indicine and African taurine) and our PCA and Admixture analyses also indicated a clear differentiation between African and European taurine breeds, supporting the selection of ancestries for the two cline analysis approaches. There is evidence for less differentiation between Asian indicine and African taurine cattle than between Asian indicine and European taurine cattle. Far fewer variants passed the filtering for sufficiently different allele frequencies between S0 and S1 when African taurine samples comprised S1 compared to European taurine S1 (8,245,114 variants were retained for European taurine S1 vs. 5,899,460 for African taurine S1). Similarly, estimated clines were steeper on average for the European taurine S1 than for the African taurine S1 (see Additional file 2: Fig. S2), which suggests greater gene flow between African taurine S1 and admixed African cattle than between European taurine S1 and admixed African cattle. Therefore, we conclude that fitting European taurine cattle as taurine ancestors captured more of the variants under selection and is thus the more appropriate approach. Nevertheless, a considerable overlap between cline estimation for both models was observed, as seen by the moderately high correlation between ln(*v*) for the two approaches and the considerable proportion of overlapping genes and regions, which has likely been driven by the common genetic background of European and African taurine cattle.

Variant type and location had a significant effect on restricted introgression: steep cline variants (SCV) were enriched within genes and the steepest clines were found for indels under both S1 approaches, indicating more restricted introgression in these functionally important variant types. Zhang et al. [12] reported similar results in a study on butterflies and hypothesised that structural variants might affect hybrid fitness and therefore contribute to reproductive isolation. Regarding SCV in coding regions, we found that 22–37% of SCV were located within annotated genes, while only ~12% of all tested variants were located within genes. Similarly, several studies have found that genomic regions with a high-density of coding or conserved elements tend to show less introgression than non-coding regions (reviewed in [69]). For the European taurine S1 approach, the steepest clines were found in missense variants, which cause changes in amino acid composition. From these observations, we can hypothesise that variants with greater phenotypic impact (indel vs. SNP, intra-genic vs. inter-genic, missense variant type) are more likely to be associated with barriers to gene flow.

While many hybrid zones are narrow relative to the dispersal distances of the organisms involved, it is now becoming clear that introgression can occur over much larger geographic distances [70, 71] and this can lead to the formation of multiple distinct geographically-based clusters isolated by pre-existing incompatibilities, and thus promote hybrid divergence and speciation [51]. Admixed African cattle occur over a vast geographical area (potentially multiple hybrid zones), and this may limit the extent to which variants can introgress across all populations, and incompatibilities can be purged from the hybrid genome. Considering all variants, without filtering for steep clines, more variants had a biased cline centre (*c* < 0.45 or > 0.55) whereas for SCV, the majority had an unbiased cline centre (*c* = 0.45 to 0.55). An unbiased cline centre (0.5) indicates that each ancestral allele does not introgress into the alternative background, i.e. the S0 allele exists primarily in samples with an h-index < 0.5 and the S1 allele exists primarily in samples with an h-index > 0.5. The fact that steep clines are mostly found at variants with unbiased cline centres is a key finding and distinguishes admixed African cattle analysed in this study from the Italian sparrow hybrid species analysed in Trier et al. [8], in which steep clines with extremely biased centre have sorted to form two distinct geographically separated boundaries. Therefore, unlike Italian sparrows, we suggest that admixed African cattle do not form a distinct hybrid taxon (characterised by steep clines with extremely biased introgression), but rather different African breeds. These breeds, especially those with low versus high hybrid indices, may be reproductively isolated from each other (characterised by steep clines with unbiased centre). This may be related to their large geographic range or other forces that favour the accumulation of incompatibilities between breeds, such as breed-assortative mating.

### The influence of recombination on allele frequency patterns

We observed that among-African breed F_ST_ and cline steepness were both significantly associated with recombination rate across the genome: higher F_ST_ (greater differentiation) and steeper clines (greater barriers to hybridization) were associated with low recombination regions, while lower F_ST_ and cline steepness estimates were associated with high recombination hotspots. Two recent studies of Heliconius butterflies have also identified genome-wide associations between introgression and recombination rate. Martin et al. [72] found a strong positive relationship between admixture levels and recombination rate in pairs of Heliconius species, such that strong reductions in introgression were concentrated in genomic regions with a low recombination rate. Furthermore, in a phylogenetic analysis of 20 Heliconius species, Edelman et al. [73] also observed that introgressed loci were underrepresented in low-recombination genomic regions. Similar patterns have also been documented in other taxa, e.g. monkeyflowers [74], swordtail fish [75], and maize [76]. In contrast to other studies, which mainly focus on low recombination regions, our study found larger differences between regions of high recombination and the rest of the genome than between regions of low recombination and other regions. However, it should be noted that the recombination data used for the comparison is based on two Holstein bulls only, and thus general application to indigenous African cattle might be limited. Additional analyses should be carried out once more recombination data (e.g. for African breeds) become available.

We also observed that among-breed F_ST_ within Africa was correlated with cline steepness: variants with the highest F_ST_ (> 0.4) had, on average, the steepest clines (Fig. 5), which is consistent with the results from a study of a contact zone between two related species of toad-headed lizards, in which Gao et al. [54] found that highly divergent regions had steeper clines and significantly lower recombination rates. Because F_ST_ is estimated without reference to ancestry, this positive relationship reveals that admixture has made a significant contribution to divergence among breeds, and this may have led to pre-existing incompatibilities (steep clines) clustering at boundaries between breeds. Selection and limited recombination, partially due to breed-specific assortative mating, may have maintained differences between African cattle of primarily indicine and taurine ancestry in many genomic regions and thus contribute to a lack of homogenization across the genome, in a similar manner to that seen for the toad-headed lizards [54].

### Molecular mechanisms underlying barriers to gene flow in hybrids

To reveal the underlying mechanisms for restricted introgression and subsequent genetic differentiation in hybrids, we evaluated the functions of the genes that harbour SCV and the genes that are located in regions with restricted introgression.

Genomic incompatibilities and reproductive isolation cause restricted gene flow. Based on recent studies, it has emerged that incompatibilities between mitochondrial and nuclear DNA play a particularly important role in generating barriers to gene flow between closely-related populations [77]. In admixed African cattle, it has been shown that while the nuclear genome reveals admixture between indicine and taurine ancestries (with indicine the dominant ancestry), the mitochondrial genome is purely taurine [78], suggesting incompatibilities between the taurine mitochondrial genome and the indicine nuclear genome [38, 39]. While we have not analysed mitochondrial data in this study, our results may still be informative on this issue as most mitochondrial proteins are encoded in the nuclear genome [79]. We found that one of the longest regions with restricted introgression for the European taurine S1 approach (see Additional file 3: Table S3; REG_eu996, BTA7 ~ 51Mb) harboured a gene (*NDUFA2*) involved in the nuclear oxidative phosphorylation (OXPHOS) system, which involves several protein complexes made up of subunits encoded by both the nucleus and mitochondria. The OXPHOS system represents a prominent example of mito-nuclear interaction and a number of OXPHOS mitochondrial genes on BTA7 were previously associated with the potential for mitonuclear incompatibility in African cattle (see Fig. 5 in McHugo et al. [80]).

The longest region with restricted introgression for the European taurine S1 approach is located around 19 Mb also on BTA7 (see Additional file 3: Table S3; REG_eu960) and includes 27 genes, which complicates their consideration as functional candidate genes. The molecular functions of these 27 genes are diverse, however the GO molecular function “DNA-binding transcription factor binding” was nominally enriched, due to three genes in the region (*PIAS4*, *DAPK3*, and *ZBTB7A*), and showed the greatest significance level (p-value = 0.004) across GO terms. The same GO term was also overrepresented in a population differentiation study of copy number variation in *Bos taurus*, *Bos indicus* and their African hybrids [81]. It is known that the compatibility of mitochondrial and nuclear DNA depends on successful mito-nuclear communication and that DNA-binding transcription factors play an important role in mito-nuclear signalling in mammals [79]. Proteins such as PIAS4 [82] or ZBTB7A [83] can inhibit DNA-binding transcription factors. Thus, our results suggest that genes encoding proteins that are involved in the inhibition of DNA-binding transcription factors are functional candidates for genes involved in genomic incompatibilities.

Other genes associated with restricted introgression identified in our study have no obvious functional link to genomic incompatibilities or reproductive isolation. However, their variants might still be subject to adaptive selection. The regions with the steepest clines under both S1 approaches were located on BTA6 and included four genes: *ODAM* (REG_eu917; ~ 85Mb) and *CRMP1, EVC* and *EVC2* (REG_afr742; ~ 103Mb). The main functions of the *odontogenic ameloblast-associated* (*ODAM*) gene are associated with tooth development and antimicrobial activity related to tooth enamel [84]. The second region, which was only identified in the African taurine S1 approach, overlapped with a region referred to as the bovine chondrodysplastic dwarfism critical region [85] with *EVC2* contributing to bone development and the occurrence of this disease [86].

The second longest region in both approaches, located on BTA14 (see Additional file 3: Table S3; REG_eu1673 and REG_afr1395; 55.7–56.3Mb), only included two genes, *EMC2* and *EIF3E*. The *EIF3E* gene is associated with oocyte development in cows [87], and has also been reported to be associated with resistance to paratuberculosis, caused by *Mycobacterium avium ssp. paratuberculosis* (MAP) infection (reviewed in [88]). Paratuberculosis has been detected in indigenous and exotic African cattle populations throughout the continent and emerges as an important zoonosis [89]. The longest region for the African taurine S1 approach was located further upstream on BTA14 and only harboured the *MMP16* gene (REG_afr1419; around 74.2–74.8 Mb). The expression of the *MMP16* gene, which also included the largest number of SCV in the African S1 approach, was altered in MAP-infected macrophages on a bovine immunologically specific cDNA microarray [90]. These findings indicate that the regions with restricted introgression on BTA14 might play a role in immunological functions, and more specifically in MAP infection. Therefore, this extended region of restricted introgression may be a consequence of alleles that are under strong environmental selection rather than reduced hybrid fertility, in line with the observation that functionally important regions of the genome tend to show reduced rates of introgression [69]. Notably, Kim et al. [32] identified extreme haplotype homozygosity and allele frequency differentiation for the N’Dama and Ankole breeds compared to other African breeds in the 58-66Mb region of BTA14.

We compared the location of the regions with restricted introgression identified in our study to the results of other studies that analysed local (variant-wise) admixture and signatures of selection in African cattle [20, 32, 33, 63] and found very few overlaps. The most relevant of these for our study is the study by Kim et al. [20] that identified regions showing excesses of indicine and taurine ancestry across the genome. A region with an excess of indicine ancestry on BTA7 identified in their study was framed by regions of restricted introgression from our analysis (REG_eu992, 1350kb downstream and REG_eu996, 250kb upstream), highlighting this region as a promising candidate subject to both adaptive and restricted introgression. The case of BTA7 and the many differences seen between our study and that of Kim et al. [20] highlight the value of the cline approach to admixture analysis. This approach separates the identification of regions associated with restricted introgression (as we have focused on in our study, potentially related to genomic incompatibilities) from the identification of regions associated with biased introgression (e.g. generated by differential selection across environments).

### Challenges and future directions of the genomic cline approach

While genomic cline analysis is an exciting opportunity to analyse patterns of introgression in hybrid populations, including indigenous African cattle, which is the subject of our study, there are limitations that complicate the interpretation of the results. The forces acting on African cattle populations, such as genomic incompatibilities and natural (adaptive) or artificial selection, are complex and may be related to each other. Bierne et al. [91] argue that endogenous factors (habitat-independent or pre-zygotic isolation) cause restricted introgression and genetic differentiation at the majority of loci while exogenous genetic incompatibilities (alleles adapted to different habitats) or natural (geographical) barriers target specific genomic regions. Indeed, despite stringent filtering, we found a relatively large number of SCV distributed across the genome, which may indicate the presence of endogenous barriers. Harrison and Larson [3] also argue that genomic differentiation for allopatric populations can be the consequence of geographical separation exclusively, and if populations became sympatric, there would be the potential for gene flow. The African cattle populations investigated in this study cover a vast geographical area and the observed signatures of restricted introgression may partly be the consequence of geographical distance and the lack of opportunity for gene flow. We propose that future studies focused on samples from smaller geographical ranges would help to control for the influence of limited opportunity for gene flow. Furthermore, more sophisticated models might be needed to disentangle the underlying mechanisms for introgression patterns since endogenous and exogenous factors may be coupled.

## Conclusions

Identification of patterns of introgression across the genome has the potential to provide important insights into reproductive barriers and environmental adaptation of both domesticated and wild species. Our study focused on indigenous African cattle, which are known to have both taurine and indicine ancestries. We showed that there are genomic regions of restricted introgression between the indicine and taurine backgrounds, which suggests the existence of genetic incompatibilities, such as mito-nuclear incompatibilities, and/or reproductive isolation between populations. Furthermore, we found that variants with a strong phenotypic impact (e.g. indels, intra-genic and missense variants) are more strongly associated with genetic barriers to gene flow, which provides important insights into the molecular mechanisms of hybridisation and restricted introgression. We also found that high F_ST_ was associated with restricted introgression, which suggests that breed differentiation in indigenous African cattle could be linked to genomic incompatibilities and reproductive isolation. This finding may have implications for the production of healthy and well-adapted crossbred cattle in various settings. A functional evaluation of genes with restricted introgression suggests that mito-nuclear incompatibilities and genes associated with fitness (e.g. resistance to paratuberculosis) could contribute to restricted gene flow in indigenous African cattle. Overall, the results and workflows from this study will inform further applications of genomic cline analyses in livestock genomics.

### Supplementary Information

**Additional file 1**: Breeds and sample sizes in ancestral and test populations. Samples were grouped into ancestry and test populations based on Admixture ancestry estimations with K = 3 for two genomic cline approaches: (1) European *Bos taurus* and Asian *Bos indicus* samples as ancestral populations S1 and S0, respectively, and all African samples as test samples (“European taurine S1”) and (2) African *Bos taurus* and Asian *Bos indicus* samples as ancestral populations S1 and S0, respectively, and the remaining African samples as test samples (“African taurine S1”). ^a^One Sahiwal sample was removed from subsequent analyses due to a proportion of indicine ancestry < 0.99. ^b^One Charolais sample was removed from subsequent analyses due to a proportion of European taurine ancestry < 0.99. ^c^One Zebu Maure sample from Table 1 was removed due to high taurine ancestry (~80% proportions of taurine ancestry whereas “zebu” are expected to have high indicine ancestry). * Breeds with samples included in both S1 and test populations.

**Additional file 2**: **Figure S1.** Variants with restricted introgression. Distribution of variants with significant cline steepness (SCV) from the genomic cline analyses for whole-genome sequence data using (a) European taurine samples as S1 ancestry and (b) African taurine samples as S1 ancestry. The strength of the statistical support for SCV (higher negative Δwaic = stronger support) is plotted along the chromosomes. **Figure S2**. Comparison of cline steepness (*v*) between approaches. The cline steepness ln(*v*) of all variants is shown for the two genomic cline approaches using European taurine or African taurine samples as S1 ancestral population. The significance of the differences (p) inferring steeper clines across all variants in the European taurine S1 was calculated by a Mann-Whitney-Wilcoxon test. The null value of ln(*v*) is zero, and positive values indicate steep clines. **Figure S3**. Bar chart of top enriched GO biological process 2021 terms for European taurine S1. The top 10 enriched terms for genes harbouring variants with significantly steep clines (input gene set) are displayed based on the -log10(p-value), with the actual p-value shown next to each term. The term at the top has the most significant overlap with the input gene set. An asterisk (*) next to a p-value indicates that the term has a significant adjusted p-value (< 0.05). **Figure S4**. Bar chart of top enriched GO biological process 2021 terms for African taurine S1. The top 10 enriched terms for genes harbouring variants with significantly steep clines (input gene set) are displayed based on the -log10(p-value), with the actual p-value shown next to each term. The term at the top has the most significant overlap with the input gene set. An asterisk (*) next to a p-value indicates that the term has a significant adjusted p-value (< 0.05). **Figure S5**. Bar chart of top enriched GO biological process 2021 terms for overlapping genes between European and African taurine S1. The top 10 enriched terms for genes harbouring variants with significantly steep clines (input gene set) are displayed based on the −log10(p-value), with the actual p-value shown next to each term. The term at the top has the most significant overlap with the input gene set. An asterisk (*) next to a p-value indicates that the term has a significant adjusted p-value (< 0.05).

**Additional file 3: Table S3**. Regions of restricted introgression. Regions of restricted introgression for the European taurine S1 approach (a) and for the African taurine S1 approach (b).

**Additional file 4: Table S4**. Association between variant effect and cline steepness (*v*). Variant effects predicted by VEP (McLaren et al. [61]) were tested for their effect on v using a linear regression (y~x) where y = ln(*v*) and x = effect category. The table shows the number of variants (n) and the estimate of the effect for ln(*v*). Higher estimated ln(*v*) indicates stronger evidence for restricted introgression.

**Additional file 5: Table S5**. Regions of restricted introgression grouped by cline centre cluster. Regions of restricted introgression grouped by cline centre cluster for the European *Bos taurus* S1 approach (a) and for the African *Bos taurus* S1 approach (b).

**Additional file 6: Table S6**. Linear regression results and multiple comparisons of means for tests of association between recombination category (hotspot, coldspot, background) and F_ST_ and cline steepness (European S1 and African S1 approaches).

**Additional file 7: Table S7**. SRA, ENA and CNGB Nucleotide Sequence Archive project accession codes for cattle samples used in this study.

## Data Availability

Additional file 7: Table S7 contains the project accession codes for cattle samples used in this study. The variant calling pipeline can be recreated using BAGPIPE (https://bitbucket.org/renzo_tale/bagpipe/).
