# Supplementary material for: Mapping restricted introgression across the genomes of admixed indigenous African cattle breeds
Source: Genet Sel Evol. 2023 Dec 14;55:91. doi: 10.1186/s12711-023-00861-8 (PMC10722721; doi:10.1186/s12711-023-00861-8)
Supplement: Supplementary file 1 — Additional file 1: Breeds and sample sizes in ancestral and test populations. Samples were grouped into ancestry and test populations based on Admixture ancestry estimations with K = 3 for two genomic cline approaches: (1) European Bos taurus and Asian Bos indicus samples as ancestral populations S1 and S0, respectively, and all African samples as test samples (“European taurine S1”) and (2) African Bos taurus and Asian Bos indicus samples as ancestral populations S1 and S0, respectively, and the remaining African samples as test samples (“African taurine S1”). aOne Sahiwal sample was removed from subsequent analyses due to a proportion of indicine ancestry < 0.99. bOne Charolais sample was removed from subsequent analyses due to a proportion of European taurine ancestry < 0.99. cOne Zebu Maure sample from Table 1 was removed due to high taurine ancestry (~80% proportions of taurine ancestry whereas “zebu” are expected to have high indicine ancestry). * Breeds with samples included in both S1 and test populations. [file 12711_2023_861_MOESM1_ESM.pdf]

Additional file 1

Tables

Table S1 Sample size of cattle breeds under investigation. Overview of cattle breeds from Africa, Asia and Europe used in analyses and their corresponding sample size (n)

Geographical origin	Breed	n
Africa	**Ankole**	7
	**Baoule**	7
	**Boran**	47*
	**Djakorre**	7
	Gourounsi	3
	**Kenana**	9
	**N'Dama**	27*
	**Ogaden**	8
	**Zebu Gobra**	7
	Zebu Maure	4
Asia	Achai	2
	Bhaghnari	2
	Cholistani	1$
	Dhanni	1$
	Gabraali	1$
	Gir	2
	HisarHiryana	1$
	Sahiwal	5
	Tharparkar	2
Europe	Angus	5
	Brown Swiss	5
	Charolais	4
	Eastern Finncattle	5
	Hereford	5
	Holstein	82*
	Jersey	5
	Limousine	4
	Scottish Highland	2
	Simmental	5
	Western Finncattle	5

* Breeds with a considerably higher sample size compared to the others were reduced to ten samples
$ Samples described in Iqbal et al. [92] were considered as 'Pakistan population' (n = 4)
African breeds highlighted in bold had at least 7 samples and were considered for F_{ST} calculation

Table S2 Breeds and sample sizes in ancestral and test populations. Samples were grouped into ancestry and test populations based on Admixture ancestry estimations with K = 3 for two genomic cline approaches: (1) European Bos taurus and Asian Bos indicus samples as ancestral populations S1 and S0, respectively, and all African samples as test samples ("European taurine S1") and (2) African Bos taurus and Asian Bos indicus samples as ancestral populations S1 and S0, respectively, and remaining African samples as test samples ("African taurine S1").

	"European taurine S1"		"African taurine S1"	
	Breed	n	Breed	N
Asian *Bos indicus*	Achai	2	Achai	2
n = 16	Bhaghnari	2	Bhaghnari	2
(S0)	Cholistani	1	Cholistani	1
	Dhanni	1	Dhanni	1
	Gabraali	1	Gabraali	1
	Gir	2	Gir	2
	HisarHiryana	1	HisarHiryana	1
	Sahiwal	4[a]	Sahiwal	4[a]
	Tharparkar	2	Tharparkar	2
European *Bos taurus*	Angus	5		
n = 54	Brown Swiss	5		
(S1)	Charolais	3[b]		
	Eastern Finncattle	5		
	Hereford	5		
	Holstein	10	NA	
	Jersey	5		
	Limousine	4		
	Scottish Highland	2		
	Simmental	5		
	Western Finncattle	5		
African *Bos taurus*			Baoule	6
n = 14	NA		Gourounsi	1
(S1)			N'dama	7
African test samples	Ankole	7	Ankole	7
n = 71 (approach 1)	Baoule	7	Baoule*	1
n = 57 (approach 2)	Boran	10	Boran	10
	Djakorre	7	Djakorre	7
	Gourounsi	3	Gourounsi*	2
	Kenana	9	Kenana	9
	N'dama	10	N'dama*	3
	Ogaden	8	Ogaden	8
	Zebu Gobra	7	Zebu Gobra	7
	Zebu Maure	3[c]	Zebu Maure	3[c]

[a] One Sahiwal sample removed from subsequent analyses due to < 0.99 proportion of indicine ancestry
[b] One Charolais sample removed from subsequent analyses due to < 0.99 proportion of European taurine ancestry
[c] One Zebu Maure sample from Table 1 was removed due to high taurine ancestry (~80% proportions of taurine ancestry whereas "zebu" are expected to have high indicine ancestry)
* Breeds with samples included in both S1 and test populations
