# Supplementary material for: Mapping restricted introgression across the genomes of admixed indigenous African cattle breeds
Source: Genet Sel Evol. 2023 Dec 14;55:91. doi: 10.1186/s12711-023-00861-8 (PMC10722721; doi:10.1186/s12711-023-00861-8)
Supplement: Supplementary file 2 — Additional file 2: Figure S1. Variants with restricted introgression. Distribution of variants with significant cline steepness (SCV) from the genomic cline analyses for whole-genome sequence data using (a) European taurine samples as S1 ancestry and (b) African taurine samples as S1 ancestry. The strength of the statistical support for SCV (higher negative Δwaic = stronger support) is plotted along the chromosomes. Figure S2. Comparison of cline steepness (v) between approaches. The cline steepness ln(v) of all variants is shown for the two genomic cline approaches using European taurine or African taurine samples as S1 ancestral population. The significance of the differences (p) inferring steeper clines across all variants in the European taurine S1 was calculated by a Mann-Whitney-Wilcoxon test. The null value of ln(v) is zero, and positive values indicate steep clines. Figure S3. Bar chart of top enriched GO biological process 2021 terms for European taurine S1. The top 10 enriched terms for genes harbouring variants with significantly steep clines (input gene set) are displayed based on the -log10(p-value), with the actual p-value shown next to each term. The term at the top has the most significant overlap with the input gene set. An asterisk (*) next to a p-value indicates that the term has a significant adjusted p-value (< 0.05). Figure S4. Bar chart of top enriched GO biological process 2021 terms for African taurine S1. The top 10 enriched terms for genes harbouring variants with significantly steep clines (input gene set) are displayed based on the -log10(p-value), with the actual p-value shown next to each term. The term at the top has the most significant overlap with the input gene set. An asterisk (*) next to a p-value indicates that the term has a significant adjusted p-value (< 0.05). Figure S5. Bar chart of top enriched GO biological process 2021 terms for overlapping genes between European and African taurine S1. The top 10 enriched terms for gen [file 12711_2023_861_MOESM2_ESM.pdf]

Additional file 2

Figures

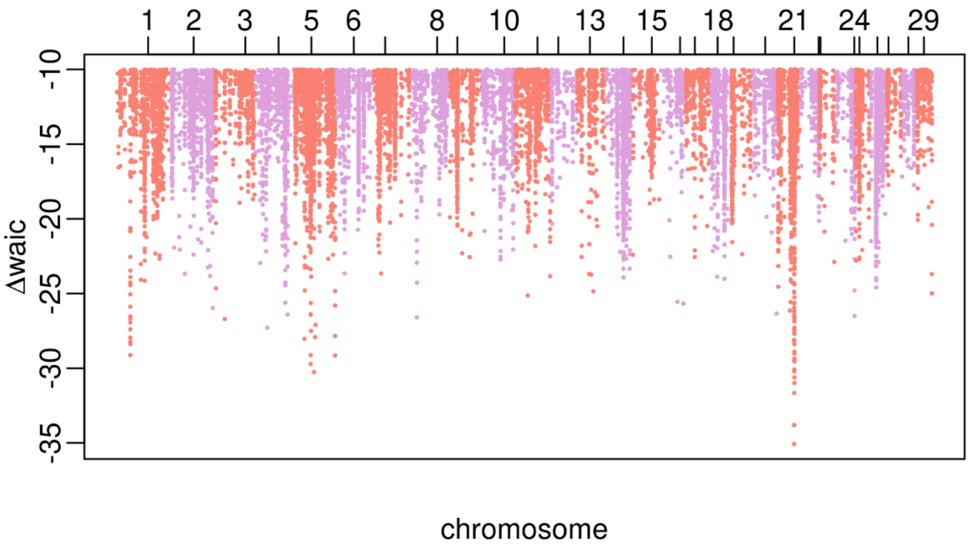

a)

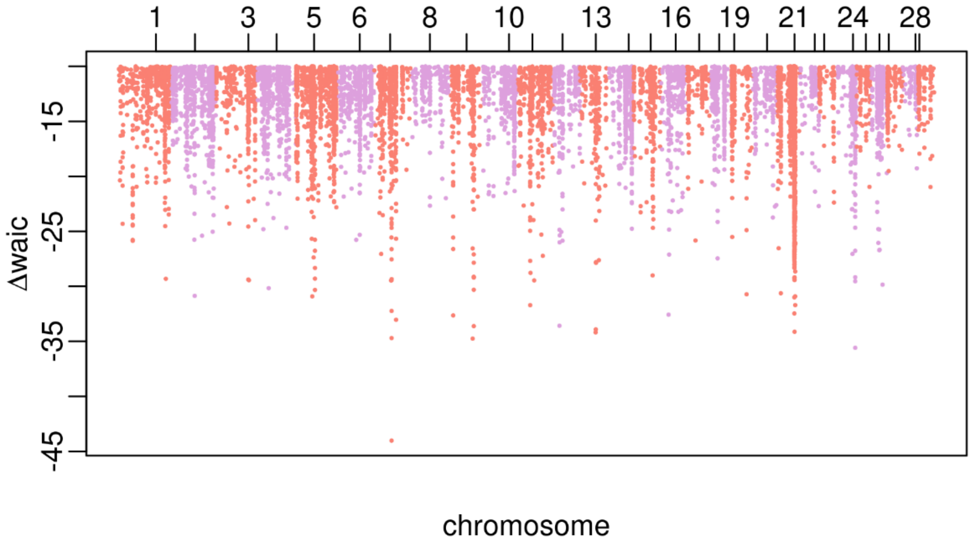

b)

Figure S1 Variants with restricted introgression. Distribution of variants with significant cline steepness (SCV) from the genomic cline analyses for whole genome sequence data using (a) European taurine samples as S1 ancestry and (b) African taurine samples as S1 ancestry. The strength of statistical support for SCV (higher negative Δwaic = stronger support) is plotted along chromosomes.

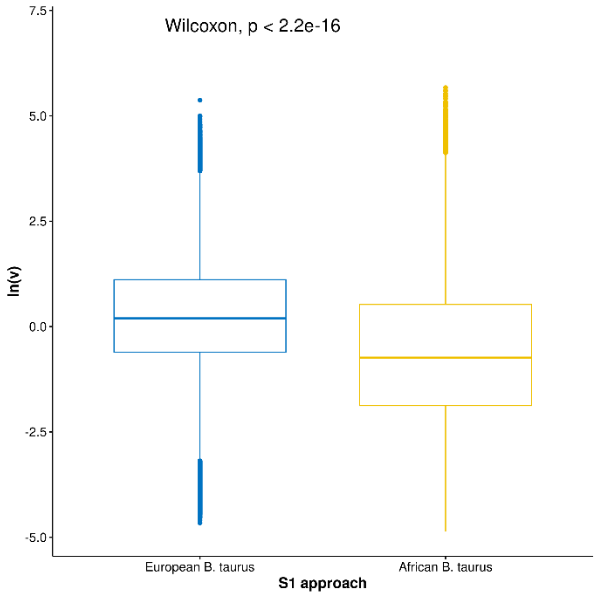

Figure S2 Comparison of cline steepness (v) between approaches. The cline steepness ln(v) of all variants is shown for the two genomic cline approaches using European taurine or African taurine samples as S1 ancestral population. The significance of differences (p) inferring steeper clines across all variants in the European taurine S1 was calculated by a Mann-Whitney-Wilcoxon test. The null value of ln(v) is zero, and positive values indicate steep clines.

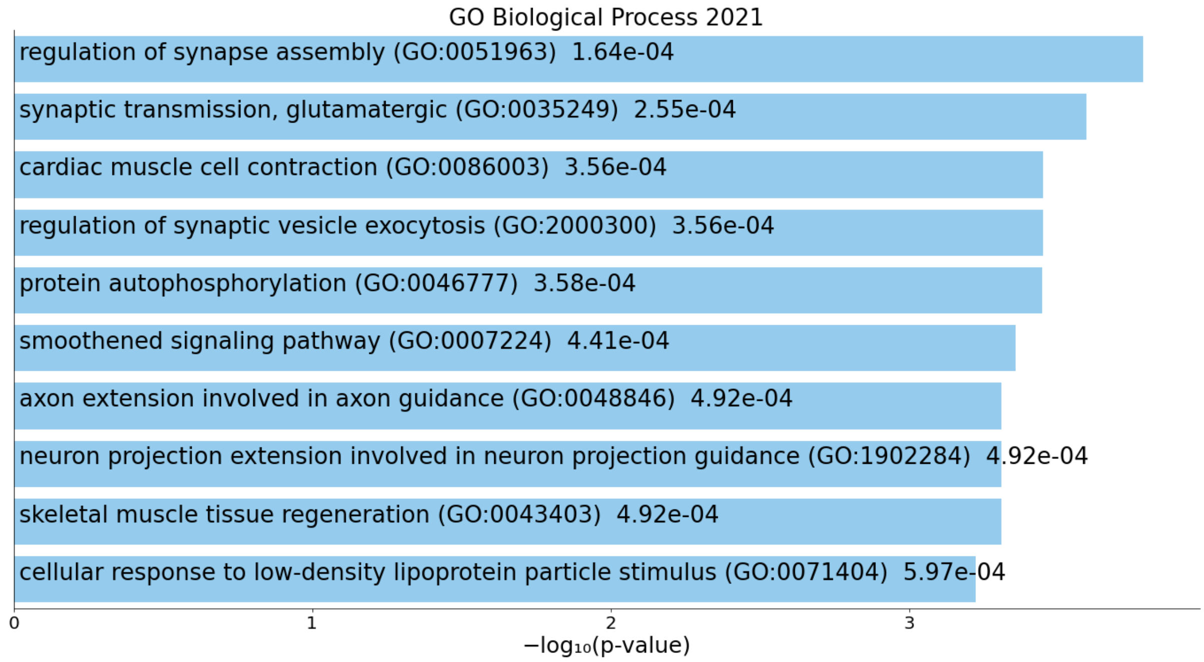

Figure S3 Bar chart of top enriched GO Biological Process 2021 terms for European taurine S1. The top 10 enriched terms for genes harbouring variants with significantly steep clines (input gene set) are displayed based on the -log10(p-value), with the actual p-value shown next to each term. The term at the top has the most significant overlap with the input gene set. An asterisk (*) next to a p-value indicates the term has a significant adjusted p-value (<0.05).

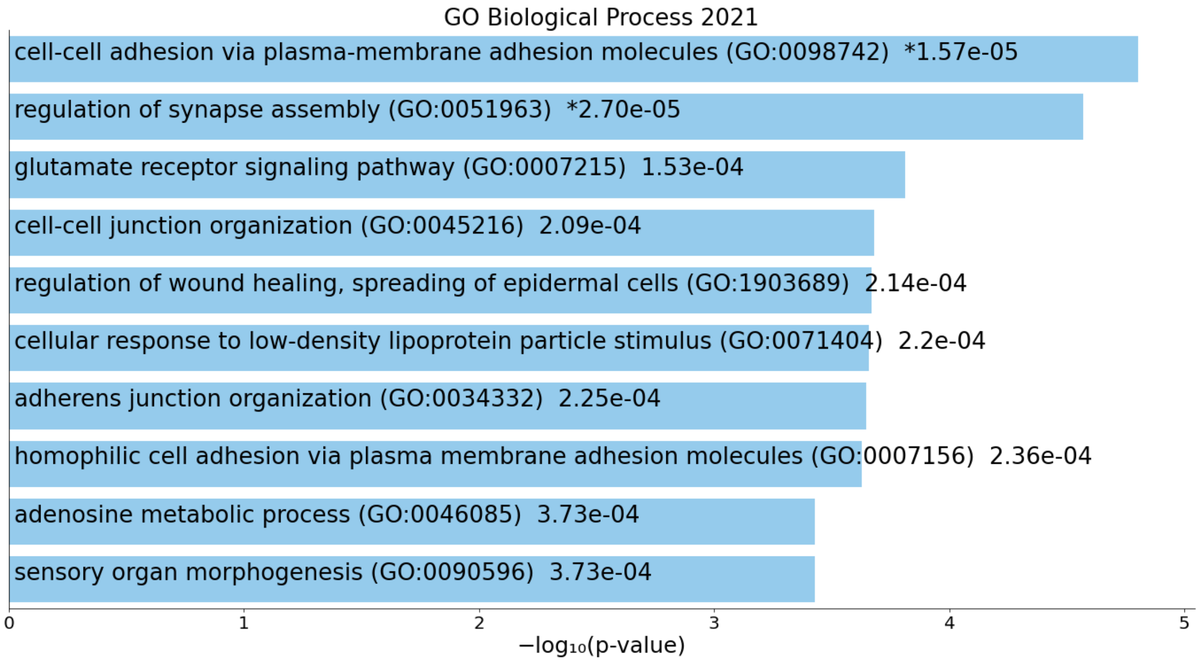

Figure S4 Bar chart of top enriched GO Biological Process 2021 terms for African taurine S1. The top 10 enriched terms for genes harbouring variants with significantly steep clines (input gene set) are displayed based on the -log10(p-value), with the actual p-value shown next to each term. The term at the top has the most significant overlap with the input gene set. An asterisk (*) next to a p-value indicates the term has a significant adjusted p-value (<0.05).

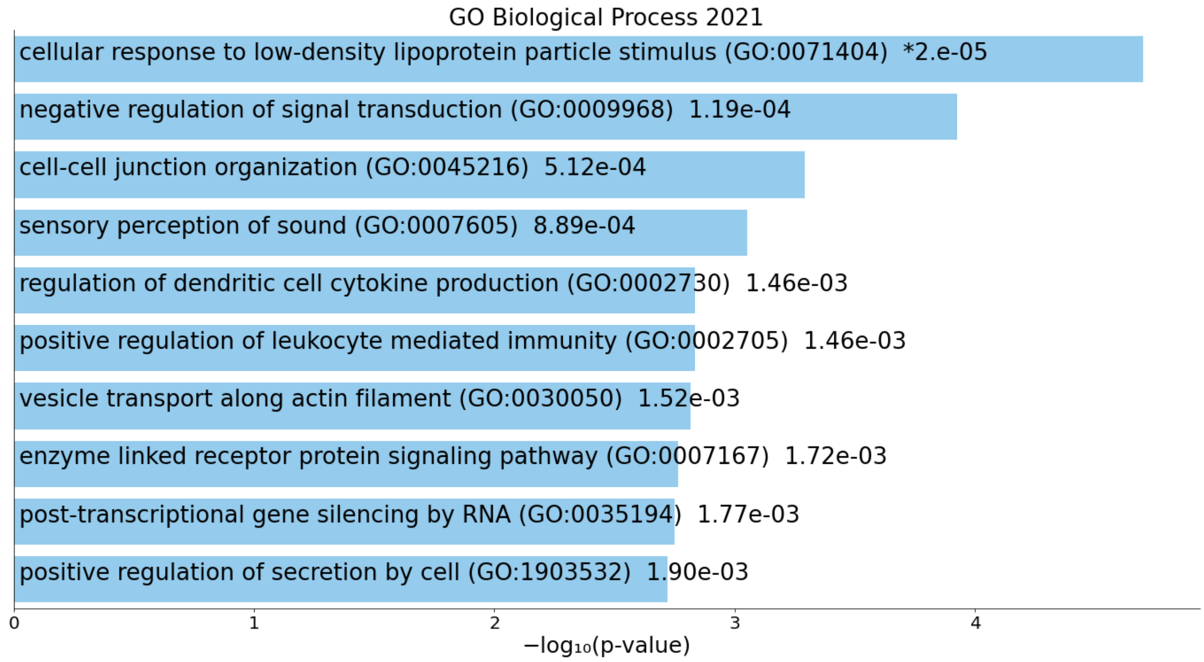

Figure S5 Bar chart of top enriched GO Biological Process 2021 terms for overlapping genes between European and African taurine S1. The top 10 enriched terms for genes harbouring variants with significantly steep clines (input gene set) are displayed based on the -log10(p-value), with the actual p-value shown next to each term. The term at the top has the most significant overlap with the input gene set. An asterisk (*) next to a p-value indicates the term has a significant adjusted p-value (<0.05).
