# Supplementary material for: Mapping restricted introgression across the genomes of admixed indigenous African cattle breeds
Source: Genet Sel Evol. 2023 Dec 14;55:91. doi: 10.1186/s12711-023-00861-8 (PMC10722721; doi:10.1186/s12711-023-00861-8)
Supplement: Supplementary file 4 — Additional file 4: Table S4. Association between variant effect and cline steepness (v). Variant effects predicted by VEP (McLaren et al. [61]) were tested for their effect on v using a linear regression (y~x) where y = ln(v) and x = effect category. The table shows the number of variants (n) and the estimate of the effect for ln(v). Higher estimated ln(v) indicates stronger evidence for restricted introgression. [file 12711_2023_861_MOESM4_ESM.pdf]

Additional file 4

Tables

Table S4 Association between variant effect and cline steepness (v). Variant effects predicted by VEP (McLaren et al. [61]) were tested for their effect on v using a linear regression (y~x) where y = ln(v) and x = effect category. The table shows number of variants (n) and the effect estimate for ln(v). Higher estimated ln(v) indicates stronger evidence for restricted introgression.

	European taurine S1		*African taurine S1*	
Effect category	n	Estimated ln(v) ± SE	n	Estimated ln(v) ± SE
3_prime_UTR_variant	21,234	0.17 ± 0.009	15,232	-0.59 ± 0.013
5_prime_UTR_variant	3,207	0.19 ± 0.023	2,329	-0.54 ± 0.032
downstream_gene_variant	10,059	0.20 ± 0.013	7,281	-0.62 ± 0.018
intergenic_variant	4,873,627	0.19 ± 0.001	3,492,131	-0.64 ± 0.001
intron_variant	2,634,414	0.21 ± 0.001	1,912,359	-0.60 ± 0.001
missense_variant	10,968	0.25 ± 0.013	7,825	-0.58 ± 0.017
non_coding_transcript_exon_variant	4,735	0.22 ± 0.019	3,535	-0.53 ± 0.026
splice_polypyrimidine_tract_variant	3,575	0.19 ± 0.022	2,594	-0.59 ± 0.030
splice_region_variant	1,899	0.18 ± 0.030	1,356	-0.55 ± 0.042
synonymous_variant	14,161	0.22 ± 0.011	10,501	-0.58 ± 0.015
upstream_gene_variant	5,500	0.20 ± 0.018	3,943	-0.60 ± 0.025
