# Supplementary material for: Mapping restricted introgression across the genomes of admixed indigenous African cattle breeds
Source: Genet Sel Evol. 2023 Dec 14;55:91. doi: 10.1186/s12711-023-00861-8 (PMC10722721; doi:10.1186/s12711-023-00861-8)
Supplement: Supplementary file 6 — Additional file 6: Table S6. Linear regression results and multiple comparisons of means for tests of association between recombination category (hotspot, coldspot, background) and FST and cline steepness (European S1 and African S1 approaches). [file 12711_2023_861_MOESM6_ESM.pdf]

Additional file 6

Tables

Table S6 Linear regression results and multiple comparisons of means for tests of association between recombination category (hotspot, coldspot, background) and F_{ST} and cline steepness (European S1 and African S1 approaches).

	hotspot fitted mean (se)	coldspot fitted mean (se)	background fitted mean (se)	Linear regression				Tukey contrasts					
				F-statistic	df	df	p-value	hotspot -coldspot		background - coldspot		background - hotspot	
								estimate (se)	p-value	estimate (se)	p-value	estimate (se)	p-value
F_{ST}	0.093 (3.6e-4)	0.102 (1.4e-4)	0.099 (2.8e-5)	420.7	2	12729577	< 2.2e-16	-0.0095 (0.00038)	<2e-16	-0.0032 (0.00014)	<2e-16	0.0062 (0.00036)	<2e-16
ln(v) (Eur)	0.081 (0.006)	0.238 (0.002)	0.197 (0.0005)	328.8	2	8245159	< 2.2e-16	-0.157 (0.0065)	<2e-16	-0.041 (0.0024)	<2e-16	0.117 (0.0061)	<2e-16
ln(v) (Afr)	-0.873 (0.008)	-0.476 (0.003)	-0.628 (0.0007)	1239	2	5899457	< 2.2e-16	-0.397 (0.0091)	<2e-16	-0.152 (0.0033)	<2e-16	0.244 (0.0085)	<2e-16
