# Supplementary material for: Mapping restricted introgression across the genomes of admixed indigenous African cattle breeds
Source: Genet Sel Evol. 2023 Dec 14;55:91. doi: 10.1186/s12711-023-00861-8 (PMC10722721; doi:10.1186/s12711-023-00861-8)
Supplement: Supplementary file 7 — Additional file 7: Table S7. SRA, ENA and CNGB Nucleotide Sequence Archive project accession codes for cattle samples used in this study. [file 12711_2023_861_MOESM7_ESM.pdf]

Additional file 7

Tables

Table S7 SRA, ENA and CNGB Nucleotide Sequence Archive project accession codes for cattle samples used in this study

Breed	Database	Project ID
Achai	CNGB Nucleotide Sequence Archive	CNP0000189
Angus	SRA	PRJNA343262
Ankole	SRA	PRJNA312138
Ankole	ENA	PRJEB39282
Baoule	ENA	PRJEB39924
Bhagnari	CNGB Nucleotide Sequence Archive	CNP0000189
Boran	ENA	PRJEB39210
Boran	SRA	PRJNA312138
Brown Swiss	SRA	PRJEB18113
Charolais	SRA	PRJNA176557
Cholistani	CNGB Nucleotide Sequence Archive	CNP0000189
Dhanni	CNGB Nucleotide Sequence Archive	CNP0000189
Djakkore	ENA	PRJEB39924
Eastern Finncattle	ENA	PRJEB28185
Gabraali	CNGB Nucleotide Sequence Archive	CNP0000189
Gir	SRA	PRJNA343262
Gourounsi	ENA	PRJEB39924
Hereford	SRA	PRJNA176557
HisarHiryana	CNGB Nucleotide Sequence Archive	CNP0000189
Holstein	ENA	PRJEB14552
Jersey	ENA	PRJNA431934
Kenana	SRA	PRJNA312138
Limousine	ENA	PRJNA343262
Limousine	SRA	PRJEB18113
Limousine	ENA	PRJNA176557
NDama	SRA	PRJNA312138
NDama	https://zenodo.org/badge/DOI/10.5281/zenodo.6855979.svg	
NDama	ENA	PRJEB36894
NDama	ENA	PRJEB39353
NDama	ENA	PRJEB39924
Ogaden	SRA	PRJNA312138
Sahiwal	ENA	PRJEB39352
Sahiwal	ENA	PRJNA379859
Sahiwal	CNGB Nucleotide Sequence Archive	CNP0000189
Scottish Highland	SRA	PRJEB18113
Simmental	ENA	PRJNA343262
Tharparkar	CNGB Nucleotide Sequence Archive	CNP0000189

Tharparkar	ENA	PRJNA379859
Western Finncattle	ENA	PRJEB28185
ZebuGobra	ENA	PRJEB39924
ZebuMaure	ENA	PRJEB39924
